# Expression rewiring and methylation of non-coding RNAs involved in rhizome phenotypic variations of lotus ecotypes

**DOI:** 10.1016/j.csbj.2022.06.001

**Published:** 2022-06-03

**Authors:** Yue Zhang, Hui Li, Xingyu Yang, Jinming Chen, Tao Shi

**Affiliations:** a CAS Key Laboratory of Aquatic Botany and Watershed Ecology, Wuhan Botanical Garden, Chinese Academy of Sciences, Wuhan 430074, China; b Center of Conservation Biology, Core Botanical Gardens, Chinese Academy of Sciences, Wuhan 430074, China; c University of Chinese Academy of Sciences, Beijing 100049, China; d Wuhan Institute of Landscape Architecture, Wuhan 430081, China

**Keywords:** Non-coding RNAs, DNA methylation, ceRNA network, Lotus rhizome

## Abstract

Non-coding RNAs (ncRNAs), including miRNAs, lncRNAs, and circRNAs, emerge as crucial components for gene regulation. *Nelumbo nucifera* (lotus), a horticulturally important plant, differentiates into a temperate ecotype of enlarged rhizomes and a tropical ecotype of thin rhizomes. Nevertheless, whether and how ncRNAs can be rewired in expression and differentially methylated contributing to adaptive divergence of this storage organ in lotus ecotypes is unclear. Herein, we study the expression behaviors and DNA methylation patterns of ncRNAs in temperate and tropical lotus rhizomes. By whole transcriptome sequencing, we found both mRNAs and lncRNAs have divergent expression patterns between ecotypes, whereas miRNAs and circRNAs tended to be accession-specific or noisier in expression. The differentially expressed ncRNAs are involved in phenotypic differentiation of lotus rhizome between ecotypes, as the genes that interacted with them in the competing endogenous RNA network are enriched in functions including carbohydrate metabolism and plant hormone signaling, being critical to rhizome enlargement. Intriguingly, ncRNA-targeted genes are less prone to show positive selection or differential expression during ecotypic divergence due to constraints from ncRNA-mRNA interactions. The methylation levels of ncRNAs generally tend to be higher in temperate lotus than in tropical lotus, and differential methylation of lncRNAs also tends to have expression changes. Overall, our study of ncRNAs and their targets highlights the role of ncRNAs in rhizome growth variation between lotus ecotypes through expression rewiring and methylation modification.

## Graphical abstract

**Figure:**
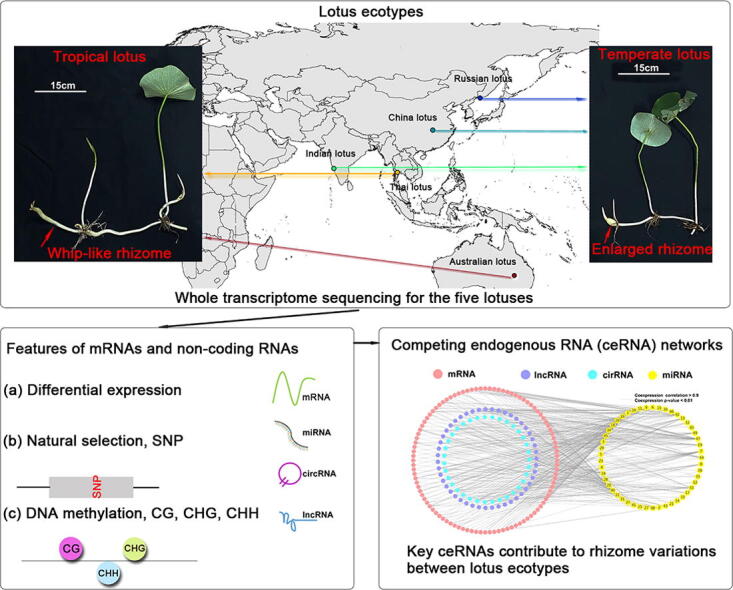

## 1 Introduction

RNAs don't only encode proteins, but also have other regulatory purposes. Apart from mRNAs, a large number of non-coding RNAs (ncRNAs) in plants were mainly grouped into three categories according to RNA length and structure: long non-coding RNAs (lncRNAs), circular RNAs (circRNAs), and microRNAs (miRNAs), and these ncRNAs play indispensable and crucial regulatory roles in plant tissue (organ) architecture and morphogenesis based on the competing endogenous RNA (ceRNA) hypothesis proposed by emerging evidence [[1], [2], [3]]. In detail, the pseudogenes, mRNAs, lncRNAs, and circRNAs could competitively take the role of ceRNAs via common miRNA response elements (MREs), and thus post-transcriptionally influence the stability and translation of target genes to modulate a broad range of plant morphogenesis and developmental processes [4]. For example, in *Populus euphratica* Oliv., morphogenesis-associated ncRNAs acted in the poplar heterophyllous morphogenesis by repressing cell division and reinforcing cell growth [[1], [5], [6]], and the lncRNA/circRNA-peu-miR396a-PeGRF and lncRNA/circRNA-peu-miR160a-PeARF regulatory (sub) networks affected the maintenance and differentiation of root apical meristems during *Populus* root development [5]. In *Arabidopsis*, an ncRNA (*HIDDEN TREASURE 1*, *HID1*) promoted red light-mediated photomorphogenesis by directly inhibiting *PHYTOCHROME-INTERACTING FACTOR 3*, while one key lncRNA, serving as a ceRNA, sequestered miR167 and regulated blue light-directed photomorphogenesis [3]. In maize, circRNAs contributed to modulating phenotypic variation by LINE1-like Element Reverse Complementary Pairs (LLERCPs) [7]. And in *Pigeonpea,* the expressivity of key genes involved in starch synthesis and sugar transportation during seed development was vastly controlled by lncRNAs and miRNAs [8]. Therefore, all these case reports suggest the crucial roles of ncRNAs via the ceRNA network in different plant organ development.

Plant rhizomes or stolons, as specialized stems and storage organs for vegetative reproduction, provide important food resources, such as potato, yam, and sacred lotus [9]. Yet, the regulatory roles of ncRNAs in shaping rhizome phenotypic variations are unclear. Sacred lotus (*Nelumbo nucifera*) or lotus, an aquatic vegetable and gardening flower widely distributed throughout Asia and Oceania with rich nutrients, including starch and anti-oxidants in rhizome [[10], [11]]. Intriguingly, in development, lotus shows adaptive phenotypic divergence according to different latitudinal environments, especially in its rhizomes and flowering time, and therefore it was further defined as two ecotypes: temperate and tropical lotus [[12], [13], [14], [15], [16], [17]]. The two ecotypes exhibit great distinction in growth behaviors and phenotypes for rhizomes. The temperate lotus is induced into dormancy under a short-photoperiod in autumn, with a short flowering period, enlarged, starch-rich, and editable rhizome, whereas the tropical one displays a long florescence period and a thin whip-like rhizome that could not withstand harsh winter in the temperate zone [18]. Although population genomics, epigenetics, proteomics, and RNA-seq studies identified key (protein-coding) genes involved in rhizome differentiation between lotus ecotypes [[15], [19], [20], [21], [22]], the expression behaviors and epigenetic regulation of different types of ncRNAs and their interactions with mRNAs in shaping the phenotypic differentiation of rhizome between these two lotus ecotypes are not clear.

To address the questions of ncRNAs in rhizome phenotypic variations and ecotypic divergence, herein we focus on how ncRNAs interact with each other and how the expression and DNA methylation differentiation of ncRNAs contributes to the phenotypic difference of rhizome between temperate and tropical lotuses. Thus, we performed whole transcriptome sequencing (WTS) on the rhizomes of both temperate and tropical lotus at the later swelling stage and analyzed the corresponding DNA methylation data we previously provided [20] to systematically investigate the expression and methylation behaviors of ncRNAs and mRNAs. Combining the WGCNA co-expression network and ceRNA regulatory network analyses, we aim to identify the key ceRNAs contributing to the rhizome variance between ecotypes, highlighting the regulatory mechanisms of ncRNAs in starch synthesis and auxin signal transduction pathways.

## 2 Material and methods

### 2.1 Plant materials

**Fig. 1:**
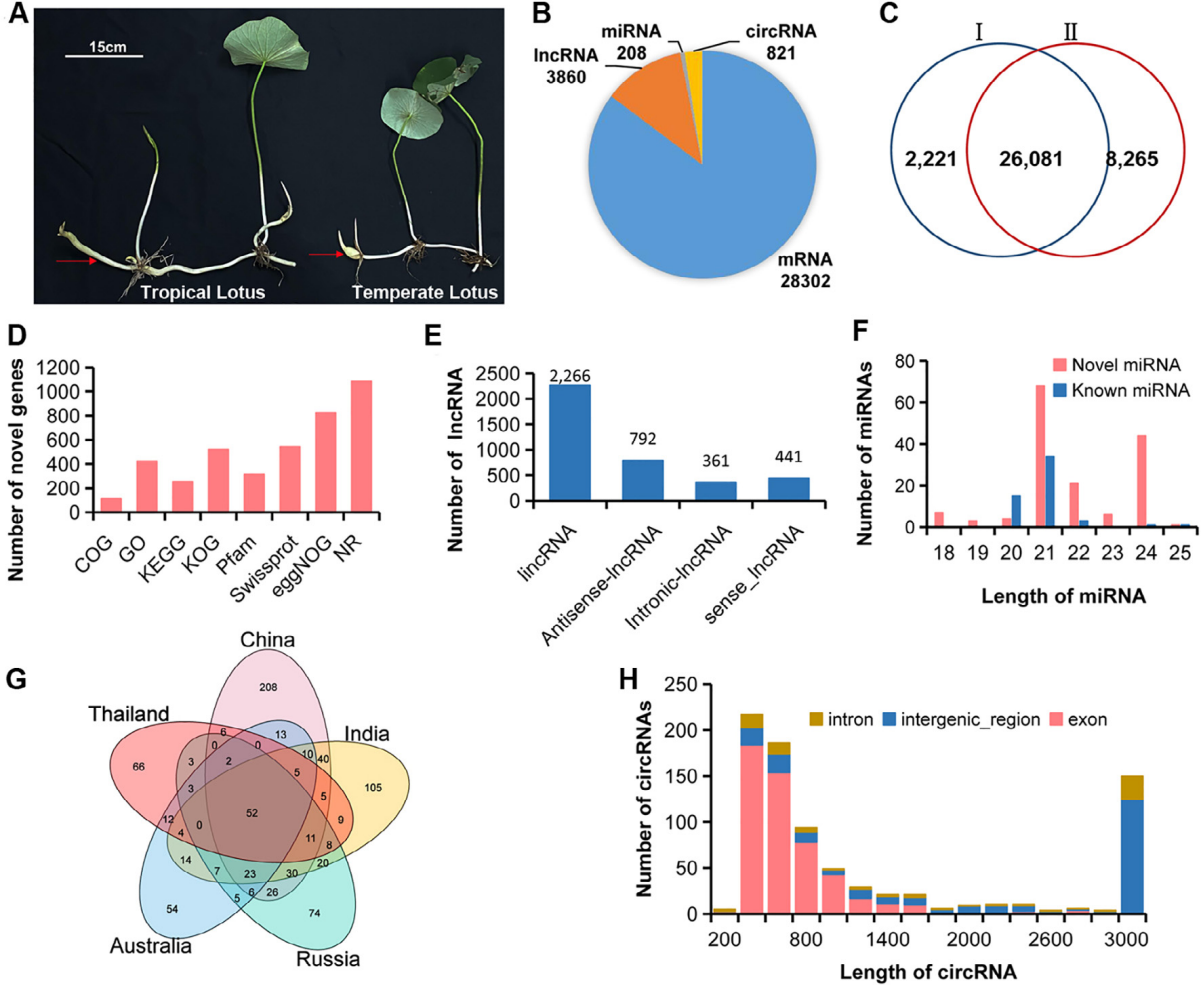
Summary of non-coding RNA detection from the whole transcriptome data of lotus rhizomes. (A) Morphological traits of two lotus ecotypes. The red arrows point to the sampling tissue. (B) Pie chart showing the number of different types of RNA. (C) Venn diagram showing the unique and overlapping mRNAs between our study (I) and the reference annotation of lotus cv. ‘China Antique’ (II). (D) Summary of functional annotation of the novel (newly identified) genes. (E) Summary of different types of lncRNA. lincRNA: long intergenic non-coding RNA. (F) Distribution of known and novel miRNAs according to their length (bp). (G) Venn diagram of the number of circRNA detected in the five lotuses. (H) Distribution of exonic circRNA, intergenic circRNA, and intronic circRNA according to their length (bp). (For interpretation of the references to color in this figure legend, the reader is referred to the web version of this article.)

The seeds of five wild lotuses, belonging to two *N. nucifera* ecotypes (temperate and tropical lotus), were collected from China, Russia, India, Australia, and Thailand. To reduce environmental variables affecting gene expression and focus on genetic influence, we performed a common garden experiment. These seeds were planted in separate vats (50 cm × 50 cm) at the Wuhan Botanical Garden of the Chinese Academy of Sciences in Hubei Province in the middle of April under the same environmental conditions. Moreover, Indian, Russian, and China lotus were grouped into the temperate ecotypes, while Thai and Australian lotus were clustered into the tropical ecotypes in our study, referring to the population structure analysis of wild accessions containing these five individuals [20]. The rhizome was sampled at the later swelling stage, according to the protocol of Yang [15] (Fig. 1A). All the tissues were sampled at 10 a.m., and then they were immediately frozen by liquid nitrogen and stored subsequently in the refrigerator at −80 °C until total transcriptome RNA extraction. All raw data were available in NCBI Sequence Read Archive (SRA, https://www.ncbi.nlm.nih.gov/Traces/sra) with accession number PRJNA510857, and the sequence files of lotus ncRNAs were deposited on the Figshare at https://doi.org/10.6084/m9.figshare.14399540.v1.

### 2.2 RNA extraction, library construction, and sequencing

Total RNAs were extracted from the rhizomes using an RNA reagent (OminiPlant RNA Kit, CWBIO, China), and incubated with RNase-free DNaseI (Thermo, Shanghai, China) for a half-hour to remove genomic DNA sequences. The degradation and contamination of RNA were monitored on 1% agarose gels also checked by the NanoPhotometer® spectrophotometer (IMPLEN, CA, USA) and Qubit® RNA Assay Kit in Qubit® 2.0 Fluorometer (Life Technologies, CA, USA), while the RNA integrity was evaluated using the RNA Nano 6000 Assay Kit of the Agilent Bioanalyzer 2100 system (Agilent Technologies, CA, USA). Finally, sequencing samples with a concentration of above 400 ng ml^−1^, RIN (RNA integrity number) values above 8, and the OD 260/280 and 260/230 ratio of above 1.8 were used to construct RNA-seq libraries.

A total amount of 1.5 μg RNA per sample was used for sequencing library construction using NEBNext® Ultra TM Directional RNA Library Prep Kit for Illumina® (NEB, USA) following the manufacturer’s manual. All the cDNA libraries were sequenced on an Illumina Hiseq 4000 platform with 150 bp paired-end reads generation. On the other hand, a total of 3 μg RNA per sample was used for the small RNA library construction. Sequencing libraries were generated using NEBNext® Multiplex Small RNA Library Prep Set for Illumina® (NEB, USA) following the manufacturer’s recommendations. The libraries of each of the five lotuses were sequenced on an Illumina Hiseq 2500 platform, and 50 bp single-end reads were generated.

### 2.3 Transcript analysis in N. Nucifera transcriptomes

After removing sequences with adapter, poly N (>10%), low-quality sequences (quality scores <20) and unpaired reads using the Trim Galore tool with default settings, clean reads were used for downstream analyses. Simultaneously, the Q20, Q30, GC content, and content size of each clean data were calculated. For mapping, all clean data in 150 bp length for each sample were mapped to the reference genome of *N.nucifera* from Nelumbo Genome Database (nelumbo.biocloud.net) [23] using HISAT2 Software v2.0.4 with a default setting values except for the parameter ‘--RNA-strandness RF’, and protein-coding genes and their FPKM (Fragments Per Kilobase of exon model per Million mapped fragments), including novel genes, were identified and estimated by using StringTie (v1.3.1) and Gffcompare software [24]. For gene functional annotation, annotations of known genes were downloaded from Nelumbo Genome Database (https://nelumbo.biocloud.net) while novel genes were searched against the NR (NCBI non-redundant protein sequences), Swiss-Prot (A manually annotated and reviewed protein sequence database), GO (Gene Ontology), KOG/COG (Clusters of Orthologous Groups of proteins), Pfam, eggNOG and KEGG (KEGG Ortholog database) databases using the BLAST (e-value <1e^−10^).

To identify differential gene (mRNA) expression between the two ecotypes, three temperate lotuses from China, India, and Russia with enlarged rhizomes were regarded as three biological replicates while two tropical lotuses from Australia and Thailand with thin rhizomes were deemed as two biological replicates. The differential expression analysis was performed using the DEseq R package [25]. The false discovery rate (FDR) was obtained by correcting for the p-value of the difference between the groups, and fold change (FC) represented the ratio of the expression amount between the two ecotypes. Genes with |log2FC| > 1.00 and FDR < 0.05 were assigned as differentially expressed genes (DEGs), and DEGs were annotated in eight databases mentioned earlier.

### 2.4 Long non-coding RNA identification, quantification, and their target gene prediction

The putative lncRNAs were identified by four different computational approaches, including CPC2 (Coding Potential Calculator), CNCI (Coding-Non-Coding Index), Pfam-scan, and CPAT (Coding Potential Assessment Tool), and the overlapped outputs from CPC2/CNCI/Pfam/CPAT, with sequence length ≥ 200nt, FPKM ≥ 0.1 and containing more than two exons were considered as reliable lncRNAs. These lncRNAs were further classified into four different types based on their genomic distributions using cuffcompare (Cufflinks suite), including lincRNA (long intergenic non-coding RNA), intronic lncRNA, anti-sense lncRNA, and sense lncRNA. This analysis was carried out in each of the five lotuses, respectively, then we merged these identified lncRNAs based on their genomic overlaps to obtain the non-redundant lncRNAs.

LncRNA expression level was calculated by FPKMs in each sample using StringTie (1.3.1). Differentially expressed lncRNAs (DELncRNAs) between temperate and tropical ecotypes (Russia, China, India vs. Thailand, Australia) were selected based on an adjusted p-value (FDR) < 0.05 and the absolute value of log2FC > 1.00 by the DEseq R package [25]. The potential *cis*-target mRNAs of DELncRNAs were predicted according to the position on the chromosome, and the adjacent genes in the 100 kb upstream and downstream of lncRNA were assigned as *cis*-targets of lncRNA. The *trans*-targets were predicted as described by the expression correlation analysis of lncRNA and mRNA, and genes with an absolute correlation value greater than 0.9 and significance p-value less than 0.01 were selected as the *trans*-target genes of lncRNA by Karl Pearson’s Coefficient.

### 2.5 Analysis of small RNA (miRNA) data

After removing the sequences smaller than 18 nt or longer than 30 nt, and reads containing ploy-N and low quality, clean small RNA reads from each of five lotuses were aligned to Silva database, GtRNAdb database, Rfam database, and Repbase database to filter transfer RNA (tRNA), ribosomal RNA (rRNA), small nuclear RNA (snRNA), small nucleolar RNA (snoRNA), other ncRNA and repeats using Bowtie v1.1.2 tool. Then, Q20, Q30, GC-content and sequence duplication levels of the clean data were calculated. Subsequently, known and novel miRNAs were identified by comparing the remaining reads against the miRBase database (https://www.mirbase.org/) by miRDeep2 v2.0.5 (-g -1 -l 250 -b 0) software [26]. The miRNA expression levels were estimated and normalized by transcripts per million reads mapped method (TPM). For differential expression analysis between temperate and tropical lotuses, the miRNAs with |log2FC| > 1.00 and FDR < 0.05 found by the DESeq R package were assigned as differentially expressed miRNAs (DEmiRNAs), and target genes of DEmiRNAs were predicted by TargetFinder with the default parameters [27], which were annotated in eight databases mentioned earlier.

### 2.6 Identification and quantification of circular RNAs

Each of the five RNA-seq datasets was also mapped to the reference genome by BWA-mem, and the CIRI tool [28] was used to predict circular RNAs (circRNAs) by scanning junction reads with paired chiastic clipping signals (PCC), paired-end mapping (PEM) and GT-AG splicing signals in the Sequence Alignment Map files (SAM). The predicted outputs were searched against the circBase database (https://www.circbase.org/) to obtain the known circRNAs and the novel circRNAs. The circRNA expression level in each sample was calculated by counting the number of junctions reads, and we standardized the circRNA expression by TPM. CircRNAs with TPM > 0 in three temperate lotuses and TPM = 0 in two tropical lotuses (TPM > 0 in two tropical lotuses and TPM = 0 in three temperate lotuses) were considered as ecotype-specific circRNAs (DEcircRNAs). The miRNA binding sites of lotus circRNAs were predicted by using the Target Finder tool [27].

### 2.7 Population genomic analysis of ncRNAs and genes between temperate and tropical lotus.

To investigate the microevolution of different lotus RNA species during ecotype divergence, we re-used our previously sequenced 19 lotus individuals, including both temperate lotuses and tropical lotuses [22]. Based on the SNPs of these two (ecotypic) populations, the nucleotide diversity (π) of each population and the genetic distance (Wright’s F-statistics, F_ST_) between these two populations were calculated by Vcftools with a sliding window size = 20 kb and step = 2 kb [29]. We further extracted the windows under the threshold of the upper 5% for F_ST_ and the lower 5% for nucleotide diversity (π_temperate_/π_tropical_ or π_tropical_/π_temperate_). Only those sliding windows that meet those thresholds were considered to be selected in temperate lotus (π_temperate_/π_tropical_) or tropical lotus (π_tropical_/π_temperate_), respectively. Gene and ncRNAs located in or overlapped with genomic regions under selection were considered as selected genes or ncRNAs.

### 2.8 Re-analysis of ecotypic DNA methylation data for non-coding RNAs

The well-annotations of methylated cytosine sites (including CG, CHG, and CHH) from whole-genome bisulphite sequencing datasets of the corresponding five lotuses investigated in this study were downloaded from our previous study [20] and used in the DNA methylation analysis for ncRNAs. The DNA methylation levels of different types of ncRNAs for each investigated region including gene body, a 2 kb fragment upstream/downstream of the transcriptional start/termination site (TSS/TTS) were independently calculated, as previously described [[20], [30]]. Besides, the differentially methylated regions between temperate (China, Russia, and India) and tropical (Australia and Thailand) lotus rhizomes were also obtained from our previous study [20] for the comparative analysis of DNA methylation of ncRNAs.

### 2.9 Construction of co-expression and ceRNA networks

To unveil how target genes were associated with rhizome enlargement of lotus, we built a gene (mRNA) co-expression network, we used a total of 76 RNA-seq samples from 13 tissues of lotus (including 17 enlarged rhizome samples and 6 whip-like rhizome samples) for building the gene co-expression network by WGCNA (weight gene co-expression network analysis) [31], which were downloaded from NCBI SRA (Table S1). The expressed genes (average FPKM > 0.1 in all samples) were filtered for constructing the gene expression matrix as the input data for WGCNA. The module eigengenes (MEs), named by random colors, were defined as the principal component and clustered sets of at least 100 genes with a similar expression. Target genes or genes regulated by non-coding RNAs were identified for each module.

To explore the functions of ncRNAs and to reveal the gene and ncRNAs interaction profile, we further constructed the ceRNA network in the lotus storage organ (rhizome) based on the following principles: 1) the number of overlapping miRNAs between ceRNAs is greater than 5; 2) Both P-value and FDR of the hypergeometric test are less than 0.01; 3) Pearson correlation coefficient of expression between ceRNAs is more than 0.99 (or less than -0.99) with p-value less than 0.01. Finally, the WGCNA co-expression network and ceRNA networks in rhizome organs were integrated and visualized using the Cytoscape tool (3.7.1) (https://cytoscape.org/).

### 2.10 Lotus SPL gene identification and phylogenetic analysis

By searching the genes against the Pfam (http://pfam.xfam.org/) and NR (https://www.biostars.org/p/154704/) database, genes containing the SBP domain and predicted as ‘squamosa promoter-binding-like protein’ were selected as NnSPL genes. 16 AtSPL and 19 OsSPL protein sequences were retrieved from the TAIR database (http://www.arabidopsis.org/) and the Rice Genome Annotation Project (http://rice.plantbiology.msu.edu/). Multiple sequence alignment of proteins for *AtSPL*, *OsSPL,* and *NnSPL* was constructed using ClustalW (https://www.genome.jp/tools-bin/clustalw) under default settings. The molecular phylogenetic tree for *Arabidopsis*, rice, and *N. nucifera* SPL gene family members was built using MEGA-X with the maximum likelihood (ML) method bootstrap replicates of 1000 [32].

### 2.11 Function and pathway enrichment analyses

We annotated target genes of ncRNAs and intersected mRNAs in ceRNA networks to elucidate the functions of differentially expressed ncRNAs. The Gene Ontology (GO) terms enriched in each gene set was analyzed by the GOseq R packages based on Wallenius non-central hypergeometric distribution with a p-value threshold of 0.05. And the Kyoto Encyclopedia of Genes and Genomes (KEGG) pathway enrichment was performed using the KO-Based Annotation System tool (KOBAS v2.0) with default parameters with a p-value threshold of 0.05.

## 3 Result

### 3.1 Identification and quantification of mRNAs and ncRNAs in lotus rhizomes

LncRNA-typed cDNA libraries in our study yielded 64.43 Gb clean reads with Q30 being above 90.98% and GC percentage from 45.48% to 49.56%, and the average mapping rate being 86% (Table 1)**.** Additionally, 37,701,758 small RNA reads were generated, and about 53.42% of the reads were mapped to the *N. nucifera* reference genome [33]. The small RNA mapping rate of the wild China lotus library was the highest (77.93%) (Table 1). Totally, 28,302 mRNAs, 3,860 lncRNAs, 208 miRNAs, and 821 circRNAs were detected in our study (Fig. 1B, Tables S2-4). We found that 26,081 out of 28,302 coding genes were previously predicted in the reference genome, and the remaining 2,221 genes represented the novel ones (Fig. 1C). Besides, a total of 1,097 novel genes were functionally annotated in eight databases (COG/KOG, GO, KEGG, Pfam, eggNOG, SwissProt, Nr) (Fig. 1D).

**Table 1:** Summary of strand-specific and small RNA-Seq libraries.

Sample	Library Type	Country	Clean Reads	N(%)	GC(%)	Q30(%)	Mapping Rate
L109	Strand-specific RNA	Thailand	87,187,410	0.00	45.48	91.09	88.79%
L4	Strand-specific RNA	China	80,596,556	0.01	49.56	93.86	93.94%
L76	Strand-specific RNA	India	96,348,388	0.01	47.03	94.58	87.48%
L81	Strand-specific RNA	Australia	86,954,960	0.00	46.15	90.98	87.91%
L99	Strand-specific RNA	Russia	39,223,526	0.00	47.54	92.07	87.95%
S109	Small RNA	Thailand	7,499,454	0.02	48.67	100.00	51.82%
S4	Small RNA	China	6,599,693	0.02	49.08	100.00	77.93%
S76	Small RNA	India	7,533,265	0.02	50.17	100.00	45.18%
S81	Small RNA	Australia	7,592,420	0.02	48.05	100.00	47.18%
S99	Small RNA	Russia	8,476,926	0.02	48.9	100.00	45.03%

Interestingly, most of lncRNAs in *N.nucifera* were lincRNA (2266, 58.70%), followed by antisense-lncRNA (792, 20.52%), intronic-lncRNA (441, 11.42%) and sense-lncRNA (361, 9.35%) (Fig. 1E, Table S2). Compared to mRNA, we found that the transcripts and open reading frame (ORF) length of lncRNAs were significantly shorter than that of mRNA, and lncRNA in lotus possessed fewer exons than mRNA (Mann-Whitney *U* test, P-value < 0.01) (Fig. S1A-C). Besides, the expression level of most lncRNA was significantly (Mann-Whitney *U* test, P-value < 0.01) lower than that of mRNA (Fig. S1D)**,** and the isoform number per lncRNA was also lower than that of mRNA (Fig. S1E). Of 208 miRNAs, 54 and 154 miRNAs were identified as known miRNAs and novel miRNAs, respectively, and 21 nt miRNA accounted for the most abundant ones (Fig. 1F, Table S3). Because our previous studies suggested that transposable elements (TEs) are an important source to give birth to novel miRNAs, particularly 24 nt miRNAs [34], the difference in TE activities between five wild lotuses might be associated with these novel 24nt miRNAs. There were 437, 343, 270, 210, and 186 circRNAs found in rhizomes from China, India, Russia, Australia, and Thailand lotus, while 52 high-quality circRNAs were detected in all five samples (Fig. 1G, Table S4). The predicted circRNAs were classified into three groups (exonic, intronic, and intergenic), and the exonic-type circRNA was the most abundant with length ranging from 400 bp to 600 bp (Fig. 1H). Moreover, the existence of the plant conserved circRNAs was validated by mapping them to the GreenCircRNA dataset using blastn with e-value < 1E-6, which confirmed a total of 295 (35.9%) conserved circRNAs in the GreenCircRNA dataset [35]. The expression level of miRNA and circRNA was normalized based on TPM. The density distribution of lotus circRNA according to ‘log10-transformed TPM’ suggested that most circRNAs were with expression level (log10-transformed TPM) from 2 to 4 (Fig. S1F), whereas miRNAs were from 1 to 3, except for China lotus (from 2 to 4) (Fig. S1G).

### 3.2 Expression patterns of ncRNAs in rhizomes between temperate and tropical lotus

**Fig. 2:**
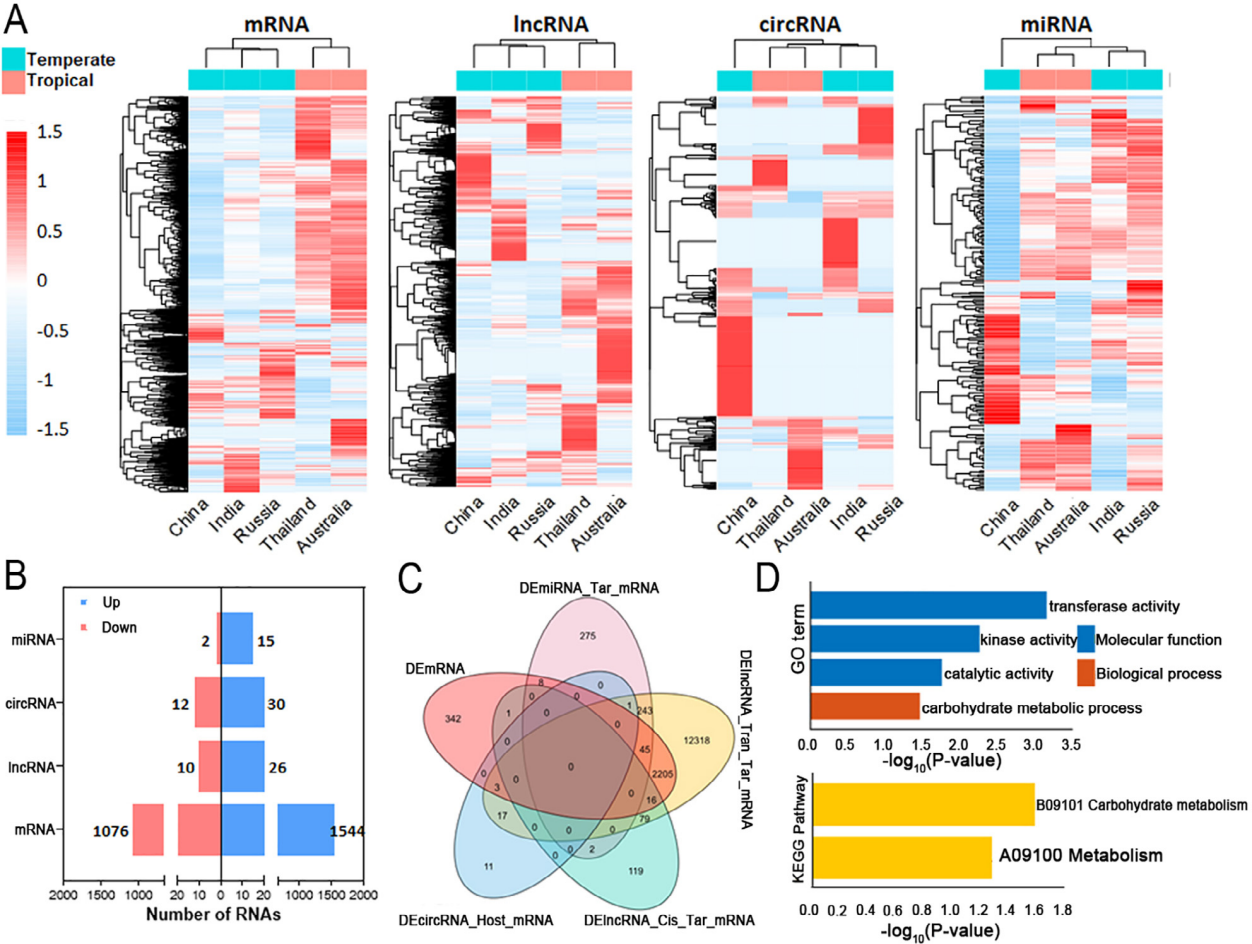
Expression patterns of mRNA, lncRNA, circRNA and miRNA in rhizomes between temperate and tropical lotus. (A) Expression profiles of different types of RNAs in the five lotus rhizomes. (B) The number of up-regulated and down-regulated mRNAs, lncRNAs, circRNAs and mRNAs in temperate lotus compared to tropical lotus. (C) Venn diagram showing the overlapped targeted mRNAs of differentially expressed ncRNAs (DEncRNAs) between the two lotus ecotypes. (D) GO and KEGG enrichment of DEmiRNA-targeted DEmRNAs in lotus rhizome. DE: differential expression between temperate and tropical lotus; Tar: targeted relationship; Tran: *trans*-targeted relationship; Cis: *cis*-targeted relationship.

To explore the expression patterns of coding and non-coding RNAs between temperate and tropical lotus rhizomes, we generated the expression profiles of different types of coding and non-coding RNAs from the rhizomes of five lotus accessions (Fig. 2A). The RNA hierarchical clustering demonstrated that there was a clear distinction between temperate and tropical lotus rhizome ecotypes in the global expression pattern. Further, we found that the expression distribution of the vast majority of mRNA and lncRNA tended to be ecotype-specific. In contrast, segregating from three temperate lotuses, circRNA and miRNA expression data in two tropical accessions were preferentially clustered together, and circRNA tended to be accession-specifically expressed (Fig. 2A). We further used the ‘accession specificity index’ (the same equation as we learned from ‘tissue specificity index’ or ‘Tau Index’) to quantify the intensity of accession bias in RNA expression [36]. We found that both line-ncRNAs (linear ncRNAs) and circRNAs were expressed in a more accession-specific manner than mRNA (Mann-Whitney *U* test, p < 0.01), except for miRNA (Fig. S2D, S2C), indicating the increased level of accession specificity for ncRNA compared with protein-coding mRNA. The ‘accession specificity index’ of circRNA was significantly higher than line-ncRNAs (Fig. S2D)**,** suggesting that the expression of these RNAs might be more conditional or accession-biased.

We observed that 2,620 mRNAs were differentially expressed (DEmRNAs) at a significant level (FDR < 0.01 and |log2FC| > 1.00) between rhizomes of two lotus ecotypes, with 1,544 DEmRNAs significantly up-regulated and 1,076 DEmRNAs down-regulated in temperate lotus, respectively (Fig. 2B, S3A). Among 3860 lncRNAs, 36 differentially expressed lncRNAs (DElncRNAs) were identified, and we found that 26 and 10 DElncRNAs were up-regulated and down-regulated in temperate lotus compared with tropical lotus (Fig. 2B, S3B). Significantly fewer DElncRNAs (36 out of 3,680, 0.93%) were found between two lotus ecotype rhizomes than DEGs (2620 out of 34,345, 7.63%) (χ^2^ test, p < 0.01), suggesting lncRNAs exhibited less conserved expression patterns within each ecotype. Besides, our results showed significantly fewer *cis*-targeted genes (17 out of 217, 7.83%) for 36 DElncRNAs (Fig. 2B) occurred differential expression between these two lotus ecotypes than *trans*-targeted genes (2269 out of 14,927, 15.20%) for that DElncRNAs (χ^2^ test, p < 0.01), suggesting that *trans*-targeting might be the major regulatory role of lotus lncRNA**.** In addition, 42 differentially expressed circRNAs (DEcircRNAs) were identified between two lotus ecotypes, with 30 and 12 DEcircRNAs up- and down-regulated in temperate lotus, respectively (Fig. 2B, S3C). Three-quarters (32/42) of these DEcircRNAs were located in the gene regions, and the intersection between 32 host genes of DEcircRNAs and DEGs was observed in the Veen diagram (Fig. 2C). Moreover, 17 differentially expressed miRNAs (DEmiRNAs) were identified, including 15 up-regulated and 2 down-regulated in temperate lotus, and of them, 4 DEmiRNAs were known miRNAs (|log2FC| > 1.00 and FDR < 0.05) (Fig. 2B, S3D). Among 574 targeted genes of these DEmiRNAs, 53 were DEGs, and we considered these DEmiRNAs as candidate miRNAs involved in lotus rhizome phenotypic difference, as well as in the regulation of the development of rhizome swelling (Fig. 2C).

To understand further the biological functions and pathways of DE RNAs, we performed functional enrichment analysis on their targeted genes. The top highly overrepresented GO and KEGG enrichment terms of DEmRNAs are carbohydrate metabolism and multiple sugar metabolisms, including starch, sucrose, fructose, and monose (p ≤ 0.05) (Figs. S5A, S6A). GO enrichment of DElncRNA targets showed that they were also enriched in the carbohydrate metabolism, while the top 5 overrepresented KEGG pathways were membrane trafficking, organismal systems, environmental adaptation, ion channels, and plant hormone signal transduction, respectively (Figs. S5B, S6B). Then, we found a growth (GO:0040007, p = 2.58E-07 with Benjamini-Hochberg method) and Transport (GO:0099977, p = 4.51E-06 with Benjamini-Hochberg method) were the most significantly enriched GO and KEGG terms for DEmiRNA targets. DEmRNAs targeted by DEmiRNAs are also significantly enriched in carbohydrate metabolism, indicating that regulation of miRNAs was important for distinct carbohydrate metabolism and plant hormone signal transduction between lotus ecotypes, which could potentially affect lotus rhizome phenotypic variations (Figs. S5C, S6C, and 2D).

### 3.3 Natural selection on ncRNAs and their associated/target genes during ecotypic divergence

Both genes or ncRNAs could be under natural selection during ecotypic adaptation, but ncRNAs were often ignored in population genomic or micro-evolutionary studies because of the extra challenge in sequencing and analysis required for identification. Here, we asked whether ncRNAs showing a different expression pattern between ecotypes are more likely to be selected during ecotypic divergence. By investigating public whole-genome resequencing data of temperate and tropical lotus, 1,335 (protein-coding) genes, 34 circRNAs, 116 lncRNAs, and 7 miRNAs were identified to be selected in temperate lotus (based on a threshold of upper 5% F_ST_ and lower 5% π_temperate_/π_tropical_) while 699 genes, 18 circRNAs, 73 lncRNAs, and 10 miRNAs were selected in tropical lotus selection (based on a threshold of upper 5% F_ST_ and lower 5% π_tropical_/π_temperate_)(see Materials and Methods), suggesting more genes, circRNAs, and lncRNAs but fewer miRNAs were selected in temperate lotus than in tropical lotus. Our results showed that the proportion of genes (7.19%) and miRNAs (8.17%) under either temperate or tropical lotus selection was significantly (χ^2^ test, p < 0.05) higher than that of lncRNAs (4.9%), but not significantly higher than that of circRNAs (6.33%) (χ^2^ test, p > 0.05), indicating more (protein-coding) genes and miRNAs were selected during the lotus ecotypic divergence than lncRNAs (Fig. S4A). Meanwhile, we found no significant (χ^2^ test, p > 0.05) differences in this proportion between lncRNAs and circRNAs, suggesting similar selection powers on lncRNAs and circRNAs during the ecotypic divergence of lotus (Fig. S4A). Besides, we found significantly fewer DEGs in genes under either temperate or tropical lotus selection than in genes without evidence of selection (χ^2^ test, p < 0.05), suggesting that the differential expression of genes between lotus ecotypes were less likely to be selected in *cis* (regulatory elements near the locus) during lotus ecotypic divergence and the expression changes are more likely contributed by *trans*-regulatory divergence (Fig. S4B). Among all classes of ncRNAs, only the lncRNAs under either temperate (5.48%) or tropical (2.59%) lotus selections showed significantly (χ^2^ test, p < 0.05) more differential expression than lncRNAs without evidence of selection, whereas other ncRNAs show no significant difference in this proportion (Fig. S4B), suggesting lncRNAs of differential expression were more likely evolved by selection in their *cis*-regulatory regions during ecotypic divergence.

Next, we asked whether genes regulated by ncRNAs are less prone to be selected as we hypothesized that genes interacting with ncRNAs are under stronger functional constraints and less likely to change during evolution. For genes being targeted or interacted with ncRNAs, intriguingly, the percentage under selection was significantly (χ^2^ test, p < 0.01) lower than the percentage without selection (Fig. S4C). This is expected given the theory that the regulatory role of ncRNAs might act as a cushion to gene expression, and therefore those genes regulated by ncRNAs might have a more stringent expression pattern during development and are more constrained by such gene-ncRNA interactions during ecotypic divergence. Indeed, by reanalyzing tissue-specificity (Tau index) from our previous study [37], we found a significantly lower tissue-specificity of expression for miRNA-targeted genes than genes without association with ncRNAs (Mann-Whitney *U* test, p < 0.01) (Fig. S4D). This suggested further the miRNA-targeted genes are with higher expression breadth in lotus tissues and under stronger functional constraints, which explains why they are constrained from selection during ecotypic divergence. Nevertheless, we found no difference in tissue-specificity between lncRNA-regulated genes and those genes without lncRNA, probably due to the fact that lncRNAs are associated with too many genes such that constraints by gene-lncRNA interactions are weak (Fig. S4D).

### 3.4 Change of methylation patterns of ncRNAs during ecotype divergence

**Fig. 3:**
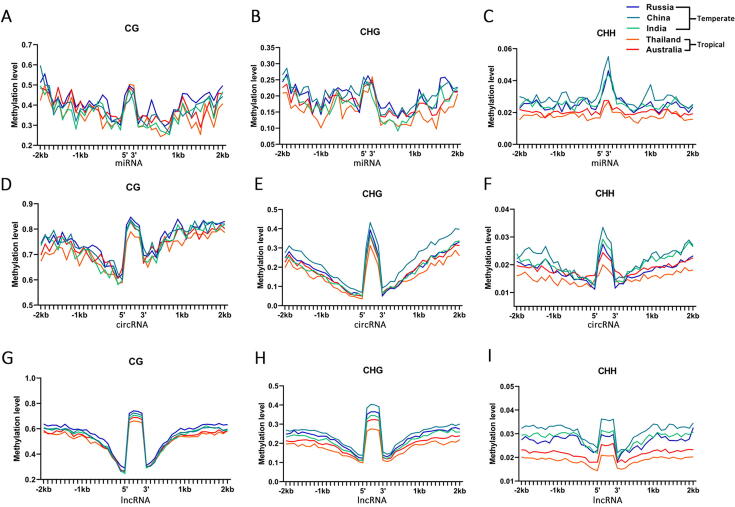
Genome-wide DNA methylation in ncRNAs from the five lotuses. (A-C) The methylation levels at CG (A), CHG (B), and CHH (C) sites in the miRNA body and surrounding regions (±2kb) from the five lotuses. (D-F) The methylation levels at CG (D), CHG (E), and CHH (F) sites in the circRNA body and surrounding regions (±2kb) from the five lotuses. (G-I) The methylation levels at CG (G), CHG (H), and CHH (I) sites in the lncRNA body and surrounding regions (±2kb) from the five lotuses.

DNA methylation of genes and flanking regions are important for gene expression. To further investigate the role of differential methylation on ncRNAs in forming ecotypic variation of rhizomes between temperate and tropical lotuses [20], here we calculated the DNA (CG, CHG, and CHH) methylation level of ncRNAs (including miRNAs, circRNAs, and lncRNAs) in five lotuses, respectively, using their corresponding bisulfite genome sequencing data (Fig. 3). First, we explored whether the methylation levels of different classes of ncRNAs are intrinsically different, we compared the DNA (CG, CHG, CHH) methylation level among miRNAs, circRNAs, lncRNAs, and mRNAs in each of the five lotuses (Fig. S7). Our results suggested that the methylation level of CG in lncRNAs was the highest in all the five lotuses, followed by miRNAs and mRNA (Mann-Whitney *U* test, p < 0.01) (Fig. S7A, Table S5). Also, the miRNAs had the highest methylation levels of CHG and CHH in five lotuses as compared to other classes of RNAs (Mann-Whitney *U* test, p < 0.01) (Fig. S7B-C, Table S5). These suggested that different RNA species have distinct general DNA methylation patterns, which were consistently observed in all the five lotuses.

Then, we compared the CG, CHG, and CHH methylation levels of temperate and tropical lotuses in the ncRNAs (body regions) and their flanking regions (2 kb upstream of the transcription start sites and 2 kb downstream of the transcription termination site). Among the comparisons between temperate lotus and tropical lotus for miRNAs, only the China lotus had a significantly (Mann-Whitney *U* test, p < 0.01) higher CHH methylation level in the RNA body region than the Thailand lotus whereas all other comparisons showed no significant differences (Fig. 3A-C, Table S5). For circRNAs, each of the three temperate lotuses had significantly (Mann-Whitney *U* test, p < 0.01) higher methylation levels of CG, CHG, and CHH in the RNA body regions of circRNAs than the Thailand lotus (tropical lotus), but slightly (Mann-Whitney *U* test, p > 0.05) higher than Australia lotus (Fig. 3D-F, Table S5). However, in the flanking regions of circRNAs, only Russia lotus had a significantly (Mann-Whitney *U* test, p < 0.01) higher CG methylation level than Thailand lotus and no significant differences were found between China, India, and Australia lotuses (Fig. 3D, Table S5). The China lotus showed a significantly (Mann-Whitney *U* test, p < 0.01) higher CHG methylation level than both Thailand and Australian lotuses in the flanking regions of circRNAs (Fig. 3E, Table S5). In addition, each of the three temperate lotuses had significantly (Mann-Whitney *U* test, p < 0.01) higher methylation levels of CHH in the flanking regions of circRNAs than the Thailand lotus, yet the Australia and Russia lotus had a similar distribution of CHH methylation (Fig. 3F, Table S5). Meanwhile, over 80% of comparisons between temperate and tropical lotuses in lncRNAs suggested that temperate lotus had a significantly (Mann-Whitney *U* test, p < 0.01) higher methylation level of CG, CHG, and CHH than tropical lotuses in both lncRNA body regions and their flanking regions (Fig. 3G-I, Table S5), similar with our previous results of DNA methylation level in genes between the two ecotype lotuses [20].

Because the differential methylation regions (DMR) of CG, CHG, and CHH between two ecotype lotuses can fuel gene expression differentiation from our previous study [20], we hypothesized this might also be true for the ncRNAs. We further compared the percentage of DMR in RNA body regions and their flanking regions between differentially expressed ncRNAs and non-differentially expressed ncRNAs (Fig. S8, Table S5). Compared to non-DElncRNAs, significantly more DElncRNAs have CHG-DMR and CHH-DMR in the RNA body regions (χ^2^ test, p < 0.05), and slightly more DElncRNAs have CG-DMR in the RNA body regions (χ^2^ test, p > 0.05) (Fig. S8G-I, Table S5). These results suggested that the differential methylation of CHG and CHH in the RNA body of only lncRNAs can fuel their expression differentiation between temperate and tropical lotus rhizomes.

### 3.5 miRNA target genes in the co-expression network of rhizome internodes

Generally, genes showing similar expression patterns are hypothesized to be functionally related. To explore the potential regulatory mechanisms of ncRNA in lotus rhizome phenotypic difference and further compare the target-gene regulation of the miRNAs among different lotus tissues, we constructed a (coding) gene co-expression network by WGCNA based on 76 RNA-seq samples from developmental stages of lotus tissues (https://nelumbo.biocloud.net) (Fig. S9A). A total of 27,224 *N. nucifera* genes were allocated to 26 modules named by random color, which clustered sets of genes with similar expression patterns among different tissues. Intriguingly, we defined the different types of rhizome internode (enlarged and whip-like) as two independent tissues (traits in WGCNA). Among all our identified WGCNA modules, 22 of them were significantly (p < 0.001) related to one of the 13 tissues, and four of them were significantly (p < 0.001) related to two of 13 tissues. Only the module ‘MEdarkorange’ was related to enlarged internode and root without significance. Notably, two WGCNA modules (2,599 genes) were significantly related to enlarged internode and two WGCNA modules (1,631 genes) were significantly related to whip-like internode, suggesting different genes are required for the different formations of internodes from the two ecotypic rhizomes. Because only miRNAs directly interact with genes (mRNAs) while lncRNAs and circRNAs are either indirectly associated with too many genes or competitively interfere with miRNA-gene interactions, we here only focused on miRNA targets in the WCGNA networks. We found that genes targeted by miRNA were allocated to different WGCNA modules, which were relevant to distinct tissues (Fig. S9B). Because our previous study revealed the tissue-specific expression of miRNAs [21] and our goal is understanding rhizome variations, we focused on the miRNA-targeted genes involved in gene modules significantly associated with rhizome internodes. Average 2.8 miRNAs regulated a total of 373 (14.35%) genes that were allocated to the enlarged internode modules, while 201 (12.32%) genes that were allocated to the whip-like internode modules were regulated by an average of 2.89 miRNAs. Also, there are more target genes of the up-regulated miRNAs in temperate lotus rhizome when comparing the target genes of up-regulated miRNAs in tropical lotus rhizome (Fig. S9C), suggesting miRNA-gene interactions were more frequent in the enlarged temperate rhizomes. Our co-expression network analyses suggested that miRNAs could play a crucial role in rhizome differentiation by targeting genes involved in the rhizome formations.

### 3.6 Construction of ceRNA regulatory network in ecotypic rhizomes

**Fig. 4:**
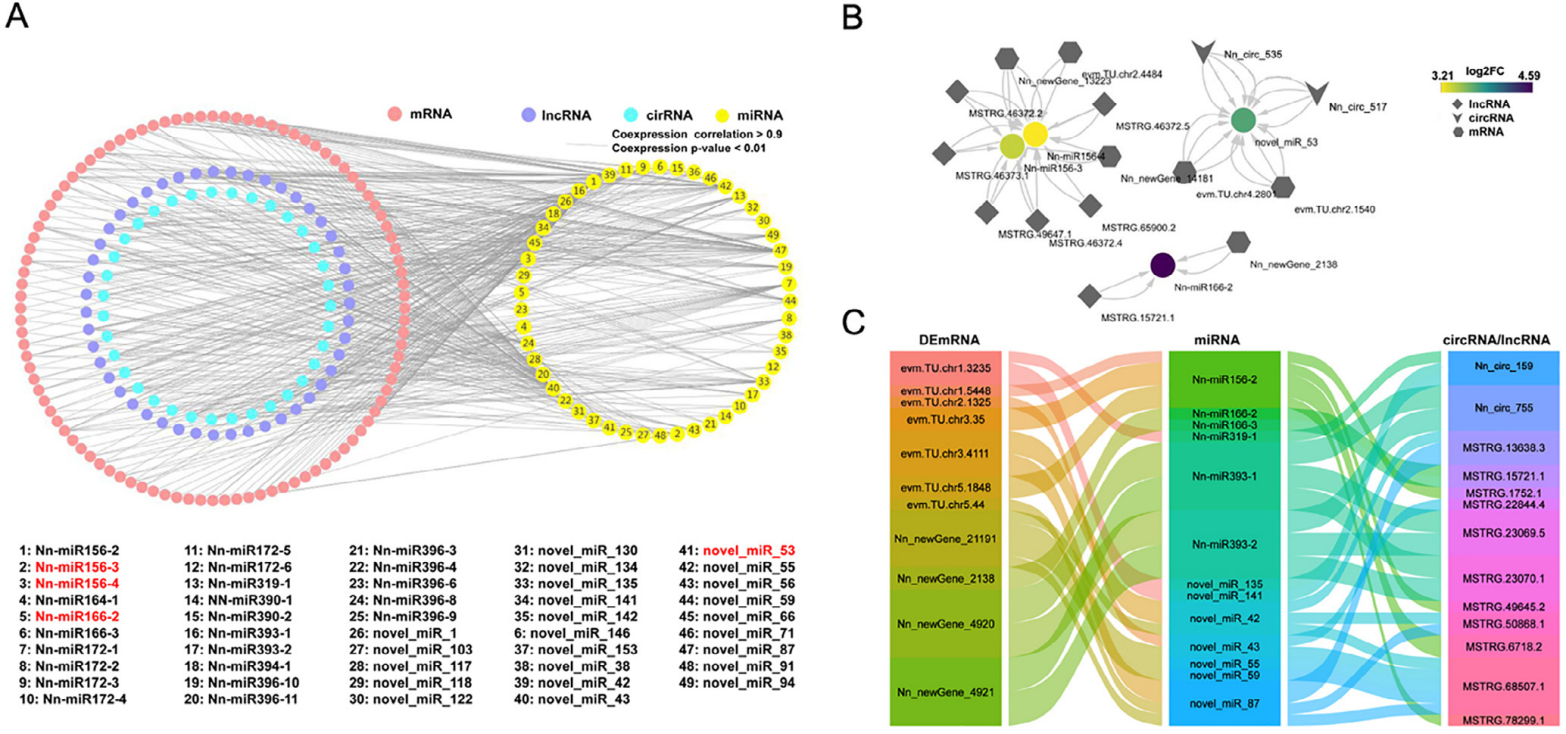
CeRNA network in lotus rhizome. (A) The global ceRNA network in lotus rhizome. The ceRNA network was constructed based on lncRNA-miRNA, circRNA-miRNA and miRNA-mRNA interactions. The edges (lines) represent sequence matching. The relationship between lncRNA or circRNA with mRNA was established via miRNA which showed a positive or negative correlation. DEmiRNAs between two lotus ecotypic rhizomes were in red. (B) Representative ceRNA sub-networks of lncRNA/circRNA-DEmiRNA-mRNA. (C) Alluvial plot showing DEmRNAs’ competing interactions with lncRNAs/circRNAs via miRNAs. (For interpretation of the references to color in this figure legend, the reader is referred to the web version of this article.)

Based on the theory of ceRNAs, the ncRNAs and mRNAs competing for the same miRNAs via MREs were selected. The ceRNA regulatory network was further constructed by integrating the expression profiles of four type RNAs to better elucidate the mechanisms underlying lotus phenotypic differentiation of rhizomes. A total of 411 ceRNA relationships were integrated from the interaction of 47 lncRNAs, 35 circRNAs, 100 mRNAs, and 49 miRNAs. Among them, 4 miRNAs were significantly differentially expressed between two lotus ecotypic rhizomes (Fig. 4A). Based on the miRNA-target relationship, we found that Nn-miR156-4 was targeted by both MSTRG.65900.2 (lncRNA) and *evm.TU.chr2.4484* (mRNA), which is responsible for coding a *trans*-acting factor (squamosa promoter-binding-like protein 14) in plant development. We assumed that the up-regulation of MSTRG.65900.2 (lncRNA) in tropical lotus rhizome likely promoted gene expression of *SPL4* by functioning as competing for endogenous RNAs (ceRNAs) for Nn-miR156-4, highlighting the pivotal roles of ceRNA networks in lotus phenotypic differentiation (Fig. 4B). In our study, a total of 24 *NnSPL* genes were identified, and the phylogenetic tree showed that these SPLs from *Arabidopsis*, rice, and *Nelumbo* were clustered into six clades, where each clade contained at least one *Arabidopsis SPL* (Fig. S10). Intriguingly, ten *NnSPL* genes were found to be targeted by the Nn-miR156 family and classified into clade 1 to clade 4, and we found that four *NnSPL* genes in these clades (*evm.TU.chr1.3376*, *evm.TU.chr1.5259*, *evm.TU.chr5.2689* and *Nn_newGene_4385*) contained no putative miR156 target site (Fig. S10), suggesting the miR156-*SPL* relationship is restricted to some subclades of the *SPL* family.

To explore the hub nodes underpinning the whole ceRNA network in lotus, 11 DEmRNAs in the ceRNA subnetwork were identified (|log2FC| > 1.00 and FDR < 0.01) and intersected with 2 circRNAs and 11 lncRNAs via 13 overlapped miRNAs based on the above interaction analysis (Fig. 4C). According to further functional inference by the closest homologs of these DEmRNAs in *Arabidopsis*, we found that they were annotated as sugar transporter ERD6-like 7 (*ERD6*), flavin-binding monooxygenase family protein (*YUCCA4*), CYP714 family protein (*CYP714A1*), HAL2-like protein (*HL*), *no apical meristem domain* transcriptional regulator superfamily protein (*RD26*), and *gamma-glutamyl transpeptidase 1* (*GGT1*) (Fig. 2D), and these genes serve critical regulatory roles in sugar metabolism and transport, plant hormone synthesis and signal transduction (auxin, gibberellin and abscisic acid) [[38], [39], [40], [41], [42]] that might contribute to rhizome enlargement.

### 3.7 ncRNAs involved in crucial biological pathways of rhizome development

**Fig. 5:**
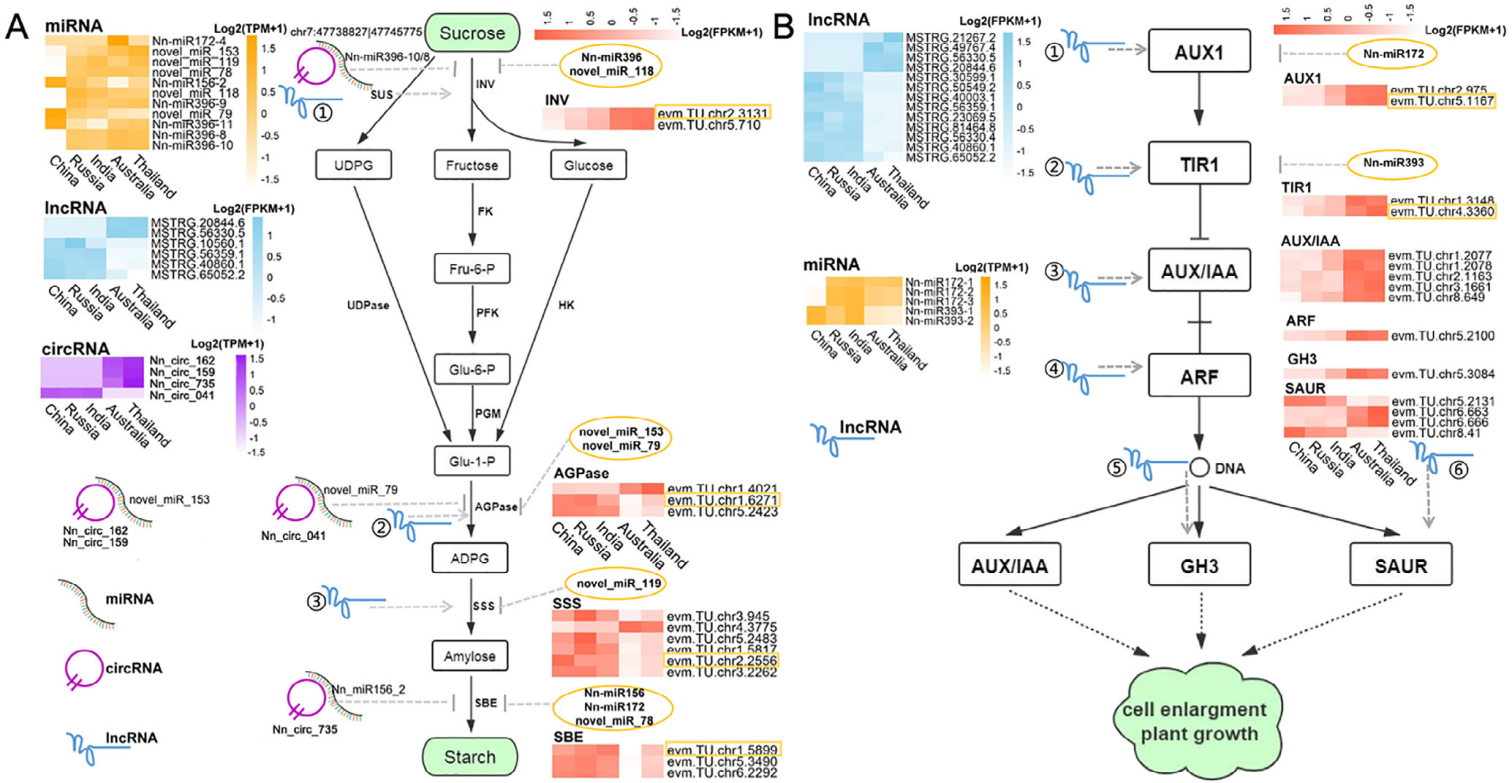
Expression of non-coding RNAs interacting with genes in starch metabolism (A) and auxin signaling (B) pathways in the five lotus rhizomes. (A) ① contains MSTRG.20844.6, MSTRG.56330.5 and MSTRG.56359.1. ② represents MSTRG.10560.1. ③ represents MSTRG.40860.1 and MSTRG.65052.2. Other DElncRNAs showing co-expression with starch-related genes are listed in Table S1. SUS: sucrose synthase, UGPase: DUP-glucose pyrophosphorylase, INV: fructofuranosidase, HK: hexokinase, FK: fructokinase, PGM: phosphoglucomutase, AGPase: AUP-glucose pyrophosphorylase, SSS: soluble starch synthase, SBE: starch branching enzyme. (B) ①-⑥represent DElncRNAs showing co-expression with auxin signaling DEmRNAs are listed in Table S2. TIR1: transport inhibitor response 1, AUX1: auxin influx carrier (AUX1 LAX family), GH3: auxin-responsive GH3 gene family, AUX1/IAA: auxin-responsive protein IAA, SAUR: SAUR family protein, ARF: auxin response factor.

To further characterize the molecular mechanisms of miRNAs and their corresponding ceRNAs underlying the intrinsic differentiation for two ecotypic lotus rhizomes, we focused on the sugar metabolism and phytohormone signaling since they were significant enrichment pathways of ncRNA targets and required for plant growth and development. Although in starch and sucrose metabolism, no obvious lncRNA/circRNA-miRNA-mRNA subnetwork was identified, we still found that the overall expression level of targeted starch-related mRNAs was significantly negatively correlated with their corresponding miRNAs in expression (Pearson *r* = −0.09 and p = 0.04) (Fig. S11A), suggesting the inhibitory roles of miRNAs in the regulation of starch genes. Also, almost half of circRNAs (384, 46.77%) and lncRNAs (1,975, 51.17%) were significantly associated with the expression of 75 ‘starch-related’ mRNAs (Fig. S11B). Based on the obtained target intersections between lncRNA-mRNA, circRNA-mRNA, and circRNA-miRNA, we observed that significantly differentially expressed starch-related genes including *invertase (INV), DP-Glucose pyrophosphorylase (AGPase), soluble starch synthase (SSS),* and *starch branching enzyme (SBE)* were targeted by Nn-miR156-2, Nn-miR172-4, Nn-miR396-10* and novel_miR_78/79/118/119/153 (Fig. 5A). Moreover, several key DEcircRNAs and DElncRNAs significantly regulating the expressions of these starch-related genes were also identified (Fig. 5B). For example, both specifically-expressed Nn_circ_735 (circRNA) and up-regulated *evm.TU.chr2.3131* (mRNA) in tropical lotus rhizome, targeted by Nn-miR396 cluster, was involved in promoting the accumulation of soluble sugars, together with MSTRG.20844.6 (lncRNA) and MSTRG.56330.5 (lncRNA). The significantly up-expressed lncRNA, MSTRG.10560.1 (lncRNA), could be significantly positively related to the expression level of one copy of *AGPase* genes in temperate lotus (Pearson *r* = 0.98 and p = 0.004), and three DEcircRNAs were also involved in ADPG synthesis through reversing their corresponding miRNA expression completely. Meanwhile, two DElncRNAs, MSTRG.40860.1 and MSTRG.65052.2 expression levels were significantly correlated with the expression of *evm.TU.chr2.2556* (mRNA) and contributed to the transition from ADPG to amylose in temperate lotus (p < 0.001). Functioning as the major determinant of the final fine physicochemical properties of the starch, one copy of *SBE* genes, *evm.TU.chr1.5899*, was significantly high-expressed in temperate lotus rhizomes, and it was targeted by Nn-miR172-4. However, up-expressed Nn_circ_735 was not significantly related to the expression of this *SBE* gene in tropical lotus rhizomes by interacting with Nn_miR156_2 (Fig. 5A, Table S6). These findings unveiled that some key ncRNAs and their corresponding mRNAs underwent significant expression alterations, and they might be associated with the difference of sugar types and starch content of rhizomes between the two lotus ecotypes.

To explore the regulatory roles of ncRNAs in the auxin signaling pathway that is important for rhizome growth, we investigated the integrated network of ncRNAs and their auxin-related targets. Firstly, we found that the expression levels of auxin-related mRNAs were weakly and negatively correlated with their corresponding miRNAs (*Pearson r* = −0.06 and p = 0.29) (Fig. S11C), and the majority of circRNAs and lncRNAs were significantly co-expressed with these genes, implying the key regulatory effects of ncRNAs on auxin signaling (Fig. S11D). Among 15 differential expressed auxin-related genes between two lotus ecotypes, 13 were up-expressed in tropical lotus rhizomes, likely being associated with stolon growth and elongation. Meanwhile, the up-regulation of Nn-miR172 and Nn-miR393 families suppressed the expression levels of *evm.TU.chr5.1167* (*auxin influx carrier*, *AUX1*) and *evm.TU.chr4.3360* (*transport inhibitor response 1*, *TIR1*), and a total of 13 DElncRNAs were found to regulate the auxin-related DEmRNA expression in each signaling step (Fig. 5B, Tabel S7). This collective evidence further implied that these ncRNAs emerged as key regulatory elements in rhizome phenotypic differentiation between temperate and tropical lotus.

## 4 Discussion

Our understanding of the intricate nature of the gene regulatory network is expanded by discovering a substantial number of non-coding RNAs (ncRNAs). Studies of ncRNAs, including lncRNAs, cicRNAs, and miRNAs in plants demonstrated their prevalence of transcription from the genomes in model and crop plants, and their diverse biological roles as super-regulators in growth, development, and response to environmental conditions [[43], [44]]. Yet, efforts on ncRNA studies have been far from adequate and confined to limited tissues from a few model plants and crops. Considering the importance of ncRNAs on gene regulation, it remains unclear the expression behaviors of ncRNAs in plant storage organs (particularly rhizomes) and how ncRNAs contribute to morphological difference and the adaptation of rhizome, for example, lotus, a traditionally ornamental flora of great economic importance in Asia [[21], [45]]. Therefore, by conducting whole transcriptome sequencing on lotus rhizome tissues in this study, we currently successfully uncovered abundant ncRNAs (lncRNAs, cicRNAs, and miRNAs) differentially expressed in rhizomes from the temperate and tropical lotus, together with DNA methylation data we also revealed the contribution of ncRNAs to the phenotypic difference between lotus rhizomes.

In our study, different species of ncRNAs have distinct attributes in structure, distribution, and expression. Consistent with previous studies, in comparison to mRNAs, lncRNAs in *N. nucifera* were shorter and presented shorter ORF, fewer exon, and lower expression levels, which is common in both animals and plants, and these characteristics might be important for their regulatory functions [[45], [46], [47]]. The majority of all 208 lotus rhizome miRNAs identified in our study were novel, which enriched the current miRNA dataset of lotus, especially for rhizomes [[21], [45]]. CircRNA numbers varied considerably in different lotus rhizomes, ranging from 186 to 437, and most of the circRNAs were exonic, which is concordant with those in *Arabidopsis* and wheat [[2], [48]]. Furthermore, the expression files of ncRNAs indicated that many ncRNAs exhibited extensive expression divergence between two lotus ecotypes, and circRNAs are more highly accession-specific or noisier in expression than linear RNA transcripts [[2], [49]]. More importantly, the biological functions of genes, targeted by differentially or accession-specifically expressed ncRNAs in rhizomes, were significantly enriched in plant growth, membrane trafficking, carbohydrate metabolism, and plant hormone signaling. This is in line with previous studies that carbohydrate metabolism and phytohormone signaling play essential roles in contributing to the distinct rhizome [[15], [22], [50]]. Furthermore, targeted genes in membrane trafficking, acting on the delivery of proteins, were found to respond to stress factors by minimizing metabolic losses in plant adaptation to their growing conditions, such as changing temperature [[51], [52], [53]].

Another crucial finding in our study is that distinct methylation patterns were found between the two lotus ecotypes and among different ncRNA species. Combining the DNA methylation dataset from our previous study [20], though we observed obvious higher CHH methylation of miRNA in temperate lotus than in tropical lotus based on methylation sites along RNA flanking and body regions, statistically, we found few significant differences in DNA methylation between temperate and tropical lotuses in both miRNA body regions and their flanking regions probably owing to weak methylation levels for CHH sites. Notably, we found generally higher DNA (CG, CHG, and CHH) methylation levels of the temperate lotus in circRNA body regions than in tropical lotuses, indicating the divergence of DNA methylation is strong in the circRNA body regions than in the flanking regions. The higher DNA (CG, CHG, and CHH) methylation levels of the temperate lotus in both lncRNAs (body regions) and their flanking regions than in tropical lotuses suggested a strong alteration of DNA methylation for lncRNAs during the ecotypic differentiation of lotus. And we should note that only differential methylation on CHG- and CHH-sites of lncRNA were found to fuel their differential expression between temperate and tropical lotus, suggesting methylation impact is stronger for lncRNAs than other ncRNA species, which is similar to the previous finding in mRNAs between lotus ecotypes [20].

The construction of a comprehensive co-expression network between ncRNAs and mRNAs greatly facilitated our understanding of the functions of ncRNAs and enriched our understanding of their regulatory mechanisms in plant growth and development [[54], [55]]. By conducting this network study, we uncovered massive lotus lncRNA-mRNA, circRNA-mRNA, circRNA-lncRNA, and circRNA-miRNA co-expressed pairs, highlighting the RNA interactions as the ‘novel’ molecular mechanisms underpinning the phenotypic divergence of rhizome between lotus ecotypes. Studies in plants demonstrated that lncRNA, circRNA, and mRNA performed crucial roles in miRNA-mediated posttranscriptional regulation of gene expression by acting as ceRNAs [[2], [45]]. In our study, the circRNA/lncRNA-miRNA-mRNA network was firstly constructed in lotus based on the ceRNA theory, and there were four key DEmiRNAs and 11 key DEmRNAs identified in this ceRNA network. Particularly, the miR156 family acts as the key regulator to modulate the expression levels of *SPL14*, which was previously found to control rice seed dormancy and seedling growth by regulating the gibberellin pathway, and the up-expression of *SPL14* targeted by DE-Nn-miR156 and MSTRG.65900.2 (lncRNA) in tropical lotus rhizome suggested that the miR156-SPL interaction might be a versatile toolbox to the rhizome growth and organ specification of lotus [56]. The number of the lotus SPLs is much larger than that in *Arabidopsis* and rice but similar to that in *Medicago truncatula*, and an equal proportion of *SPL* genes contained target regions of miR156 in three species indicated that the conservation of plant miR156-mediated posttranscriptional regulation [[57], [58]]. These results could provide an important clue for the further interpretation of the molecular function of *NnSPLs* and their regulation in lotus rhizome development and differentiation. Notably, the homologous annotation of ceRNA DEmRNAs implied that some ceRNA interactions, including circRNA-miRNA-DEmRNA and lncRNA-miRNA-DEmRNA, were involved in sugar metabolism and transport, plant adaptation to the environment, and plant hormone pathways, which were considered as key ncRNA-mRNA interactions to the rhizome phenotypic specificity between lotus ecotypes. Other than ceRNA networks in the rhizome, our study also unveils the global WCGNA network containing miRNA targets based on diverse lotus tissues, which further suggested that target genes of miRNAs are involved in rhizome differentiation between the two lotus ecotypes. Further steps towards developing relevant functional genetic experiments of these ceRNAs are important to verify their specific roles in the lotus.

Another focus of ncRNA-mRNA interactions in our study is sugar metabolism and auxin signaling pathways because the transcriptional change of protein-coding genes in these pathways is involved in the development of lotus rhizome [[15], [37], [59]]. Through regulating mRNAs from genes in different biological pathways, different ncRNAs play crucial roles in shaping plant phenotypic diversity. Evidence has shown that transgenic expression of miR156 in switchgrass has been reported to increase starch content [60], and overexpression of miR156 up-regulated miR172 target genes by repressing miR172 in grasses [61]. In our study, the regulatory effects of miRNA156/172 on *SBE* genes in lotus on starch synthesis in temperate lotus rhizome are consistent with that in those previous studies, suggesting this conserved regulatory relationship among plant species. Another conserved miR396 family was found to participate in starch metabolism by targeting the *INV* gene, and this might be a novel function of miR396s since the miR396-*GRF* (growth-regulating factor) regulatory network was previously found to be involved in regulating starch accumulation during grain development as reported in rice and maize [[62], [63]]. Importantly, because of the role in cell diversion and growth, auxin signaling also has been recognized as the causal process in storage organ formation (tuberization) of the potato in several previous studies, and miR394 in sweet potato controlled the formation and (or) development of storage root by regulating this pathway, which is slightly different from our results that miRNA393/170 was the main regulators of lotus auxin signaling pathway [[64], [65]]. In line with those findings, up-expressed Nn-miR393 also inhibited *TIR1* in temperate lotus, suggesting that the Nn-miR393/*NnTIR1* interaction might degrade the AUX/IAA repressor and concomitantly repress auxin signaling pathway in lotus [66]. Besides, miR172 and miR156 were found to be involved in potato tuber formation, and considering the regulatory functions of Nn-miR172 in both IAA signaling and starch metabolism, we also postulated that miR172 might participate in lotus rhizome morphologic difference in a complex manner by interacting with essential DElncRNAs and DEcircRNAs. Intriguingly, one copy of lotus AUXIN RESPONSE FACTORS (*ARFs*) (evm.TU.chr5.2100) was significantly up-expressed in tropical lotus rhizome but was not targeted by Nn-miR160, which targets four only lotus ARF genes. The findings of lotus miR160/*ARFs* suggested their specific roles in rhizome morphological differentiation, which seems different from that in root and shoot growth [67].

## Declaration of Competing Interest

The authors declare that they have no known competing financial interests or personal relationships that could have appeared to influence the work reported in this paper.
