# Supplementary material for: Expression rewiring and methylation of non-coding RNAs involved in rhizome phenotypic variations of lotus ecotypes
Source: Comput Struct Biotechnol J. 2022 Jun 3;20:2848–60. doi: 10.1016/j.csbj.2022.06.001 (PMC9193371; doi:10.1016/j.csbj.2022.06.001)
Supplement: Supplementary data 1 [file mmc1.docx]

**Expression rewiring and methylation of non-coding RNAs involved in rhizome phenotypic variations of lotus ecotypes**

**Short title:** ncRNAs in rhizomes of lotus ecotypes

Yue Zhang1,2,3,┼,Hui Li1,2,3,┼, Xingyu Yang4, Jinming Chen1,2*, Tao Shi1,2*

1CAS Key Laboratory of Aquatic Botany and Watershed Ecology, Wuhan Botanical Garden, Chinese Academy of Sciences, Wuhan 430074, China

2Center of Conservation Biology, Core Botanical Gardens, Chinese Academy of Sciences, Wuhan 430074, China

3University of Chinese [Academy of Sciences, Beijing 100049,](mailto:jmchen@wbgcas.cn) China

4[Wuhan Institute of](mailto:shitao323@wbgcas.cn) Landscape Architecture, Wuhan 430081, China

┼ Yue Zhang and Hui Li contribute equally to the manuscript

*Corresponding authors:Jinming Chen (**jmchen@wbgcas.cn)** and Tao Shi

# (shitao323@wbgcas.cn)

**Supplementary figures**


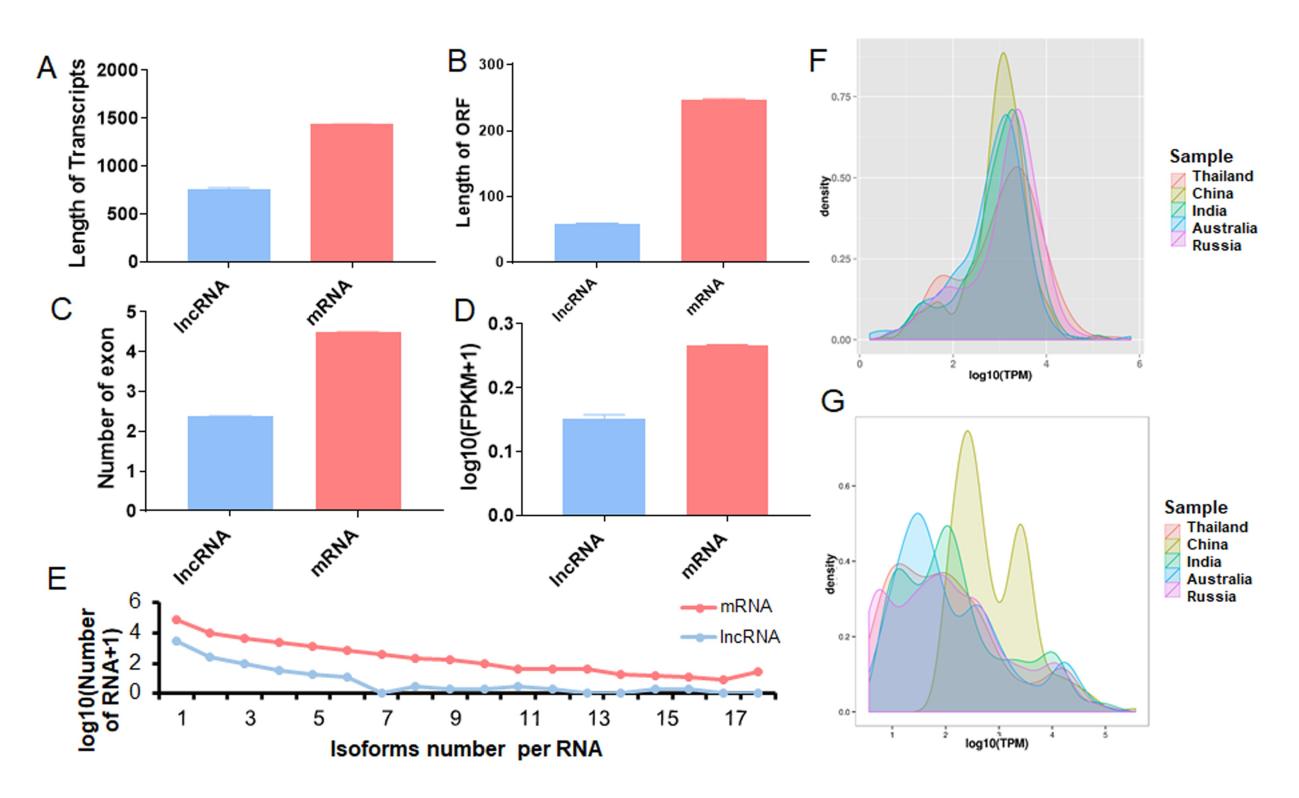


**Figure S1**. **Summary of different types of RNAs in our study.** (A-E) Comparison of lncRNA and mRNA in transcript length (A), ORF length (B), exon number (C), FPKM value (D) and isoform number (E). (F) and (G) represent TPM density distribution of circRNAs and miRNAs, respectively.


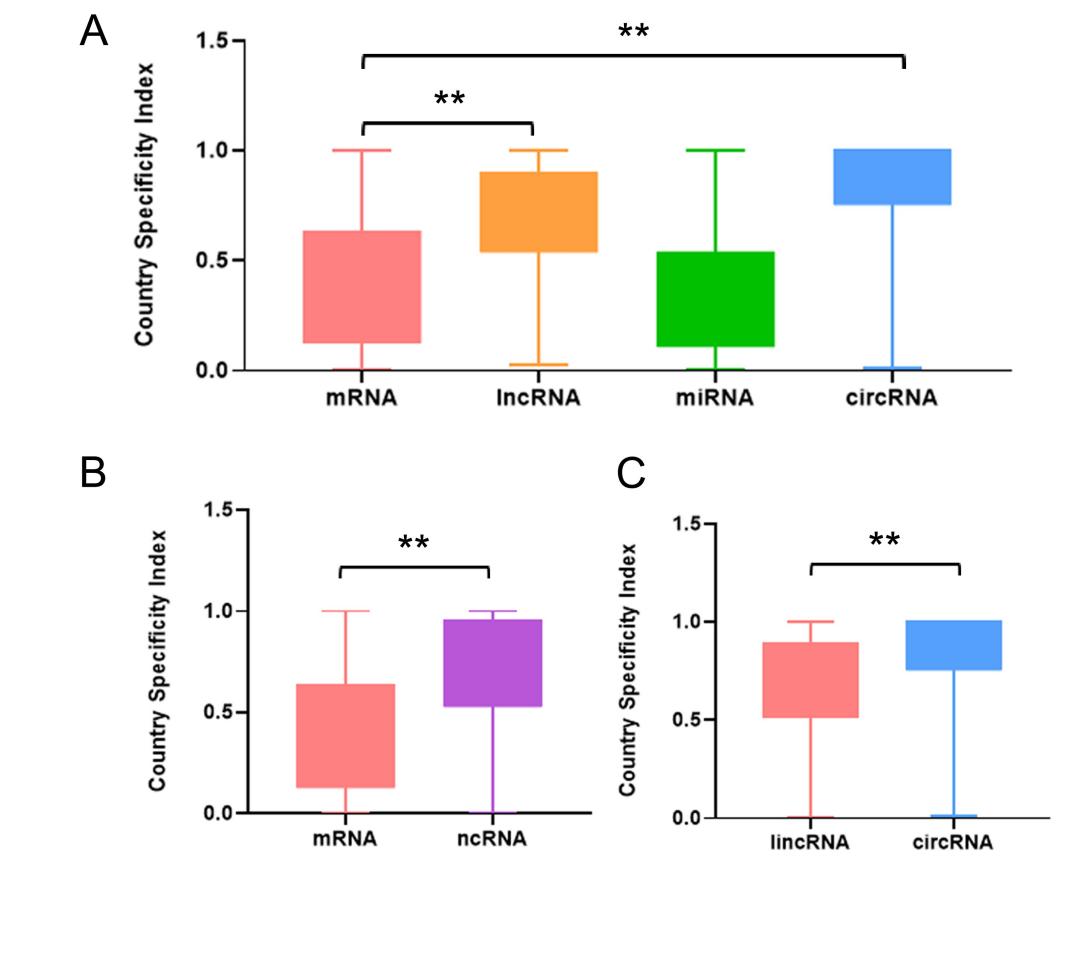


# Figure S2. Characteristics of expression specificity among different types of RNAs in our study.

1. Comparison of ‘country specificity index’ between two of the four types of RNAs.
2. Comparison of ‘country specificity index’ between mRNAs and ncRNAs.
3. Comparison of ‘country specificity index’ between line non-coding RNAs (line- ncRNAs) and circRNAs

The significance was tested by Mann-Whitney *U* test, **means p-value <0.01.


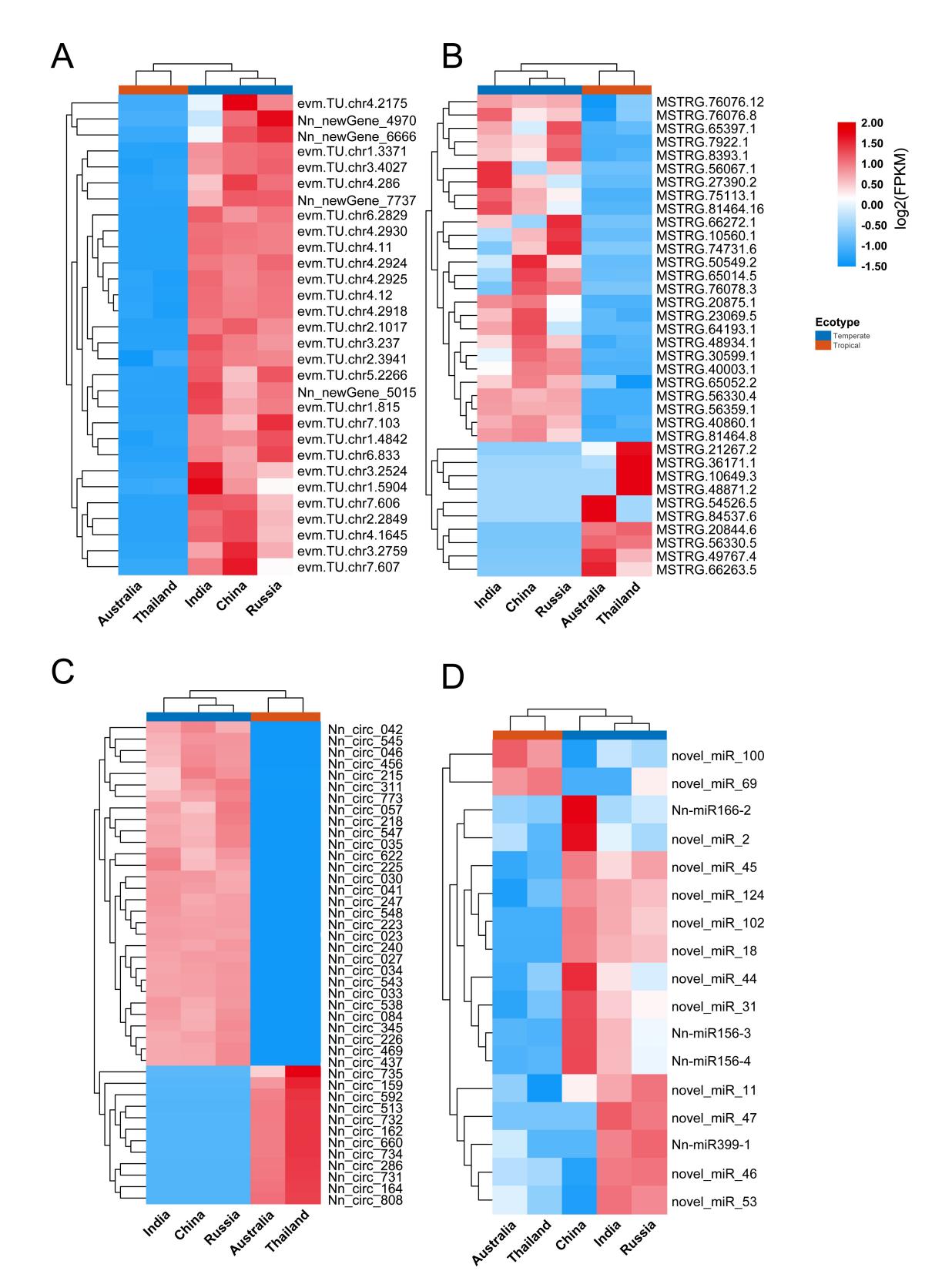


**Figure S3**. **Heatmap of the most differentially expressed mRNAs (A), lncRNAs (B), circRNAs (C) and miRNAs (D) between two lotus ecotypes.** Note that top 30 most differentially expressed mRNAs are shown.


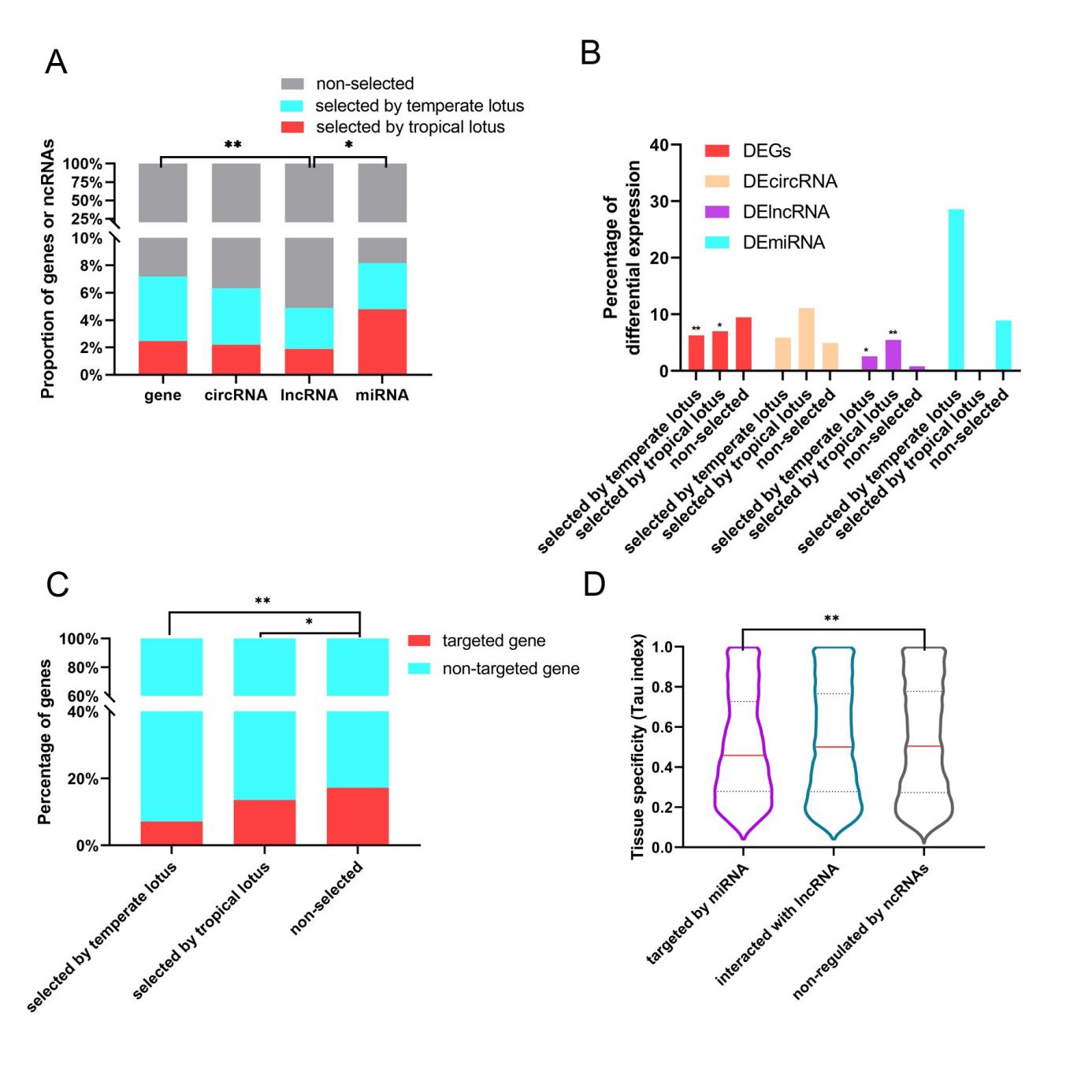


# Figure S4. The coding and non-coding RNAs were selected in temperate or tropical lotus.

- 1. Proportion of genes or ncRNAs under temperate lotus selection, tropical lotus selection or non-selection. The significance was calculated by chi-square test, ‘**’ means p < 0.01 **.**
  2. Percentage of differentially expressed genes (DEGs), circRNA (DEcircRNA), lncRNA (DElncRNA), and miRNA (DEmiRNA) in selected or non-selected genome regions. The significance was calculated by chi-square test, ‘*’ means p < 0.05.
  3. Percentage of genes are targeted by ncRNAs in selected or non-selected genes. The significance was calculated by chi-square test, ‘*’ means p < 0.05 and ‘**’ means p < 0.01.
  4. Distribution of tau index in genes that were targeted by miRNA, interacted with lncRNAs and without interaction with any ncRNA. The significance was tested by Mann- Whitney *U* test, ‘**’ means p < 0.01.


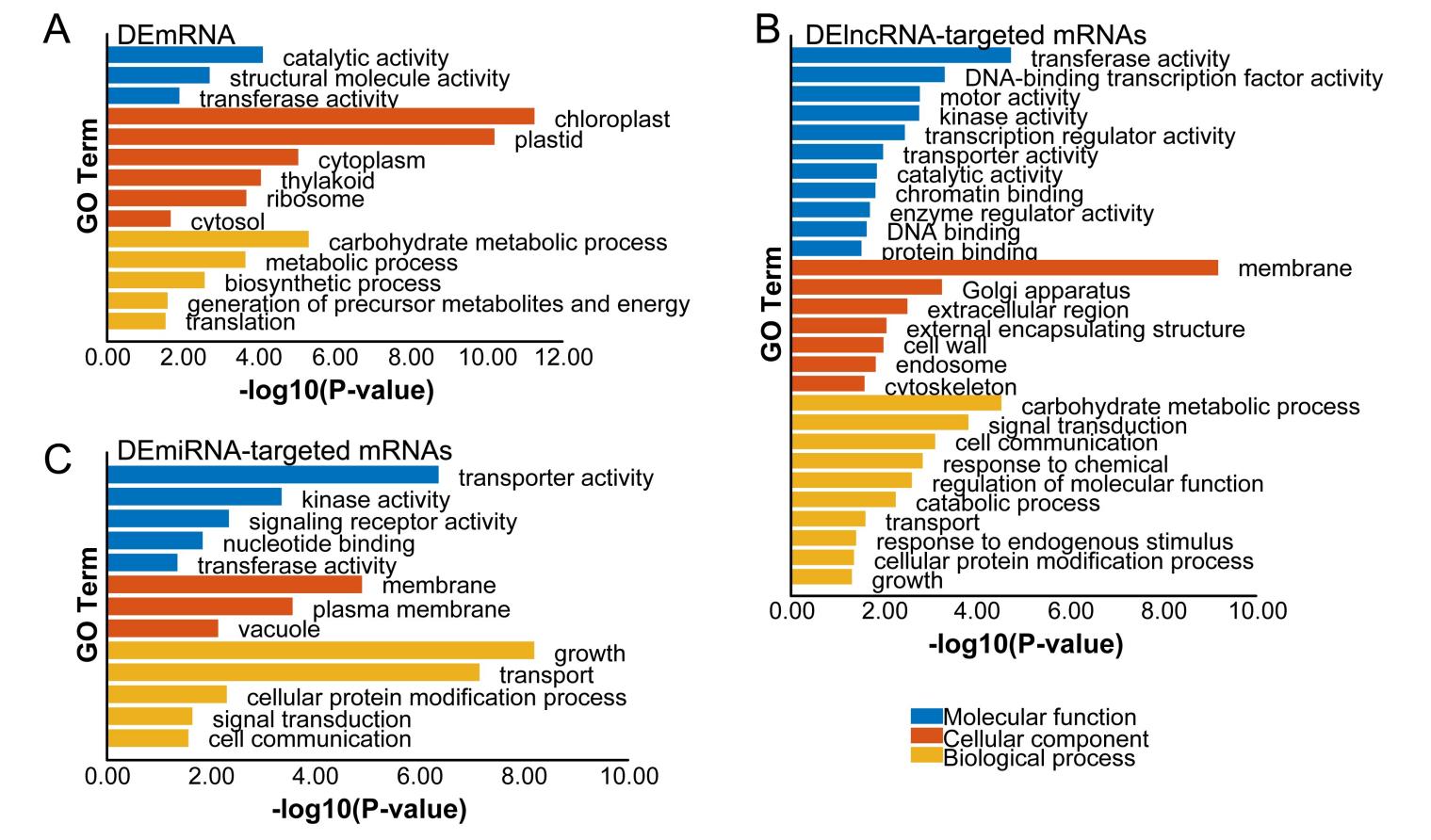


# Figure S5. GO enrichment of differentially expressed mRNAs (A), targeted mRNAs of differentially expressed lncRNAs (B), and targeted mRNAs of differentially expressed miRNAs (C).


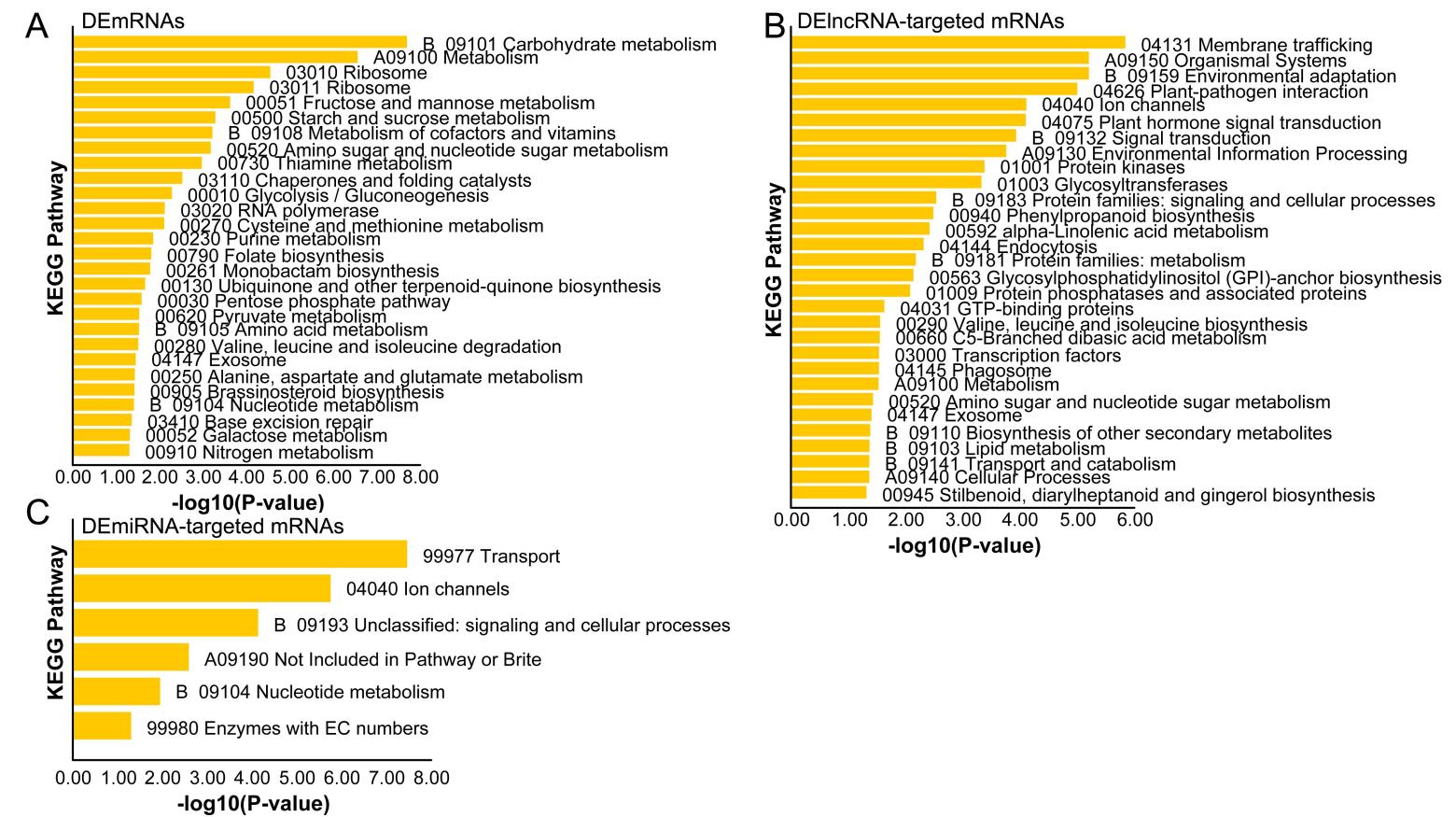


**Figure S6. KEGG enrichment of differentially expressed mRNAs (A), targeted mRNAs of differentially expressed lncRNAs (B), and targeted mRNAs of differentially expressed miRNAs (C).**


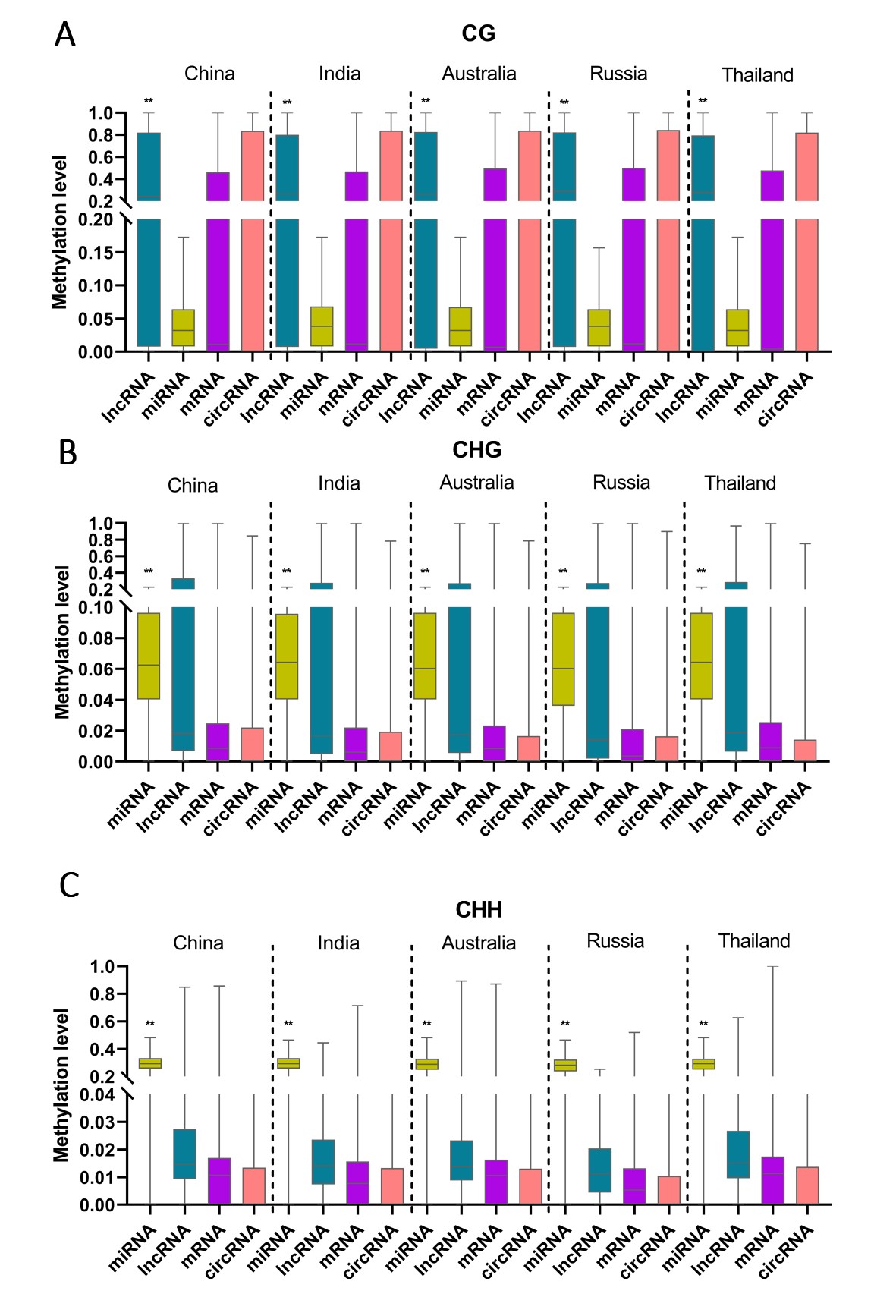


**Figure S7. The methylation level of CG (A), CHG (B), and CHH (C) in ncRNAs (including miRNA, circRNA, and lncRNA) and mRNAs for the five lotuses.**

The significance was tested by the Mann-Whitney *U* test, **means p-value <0.01.


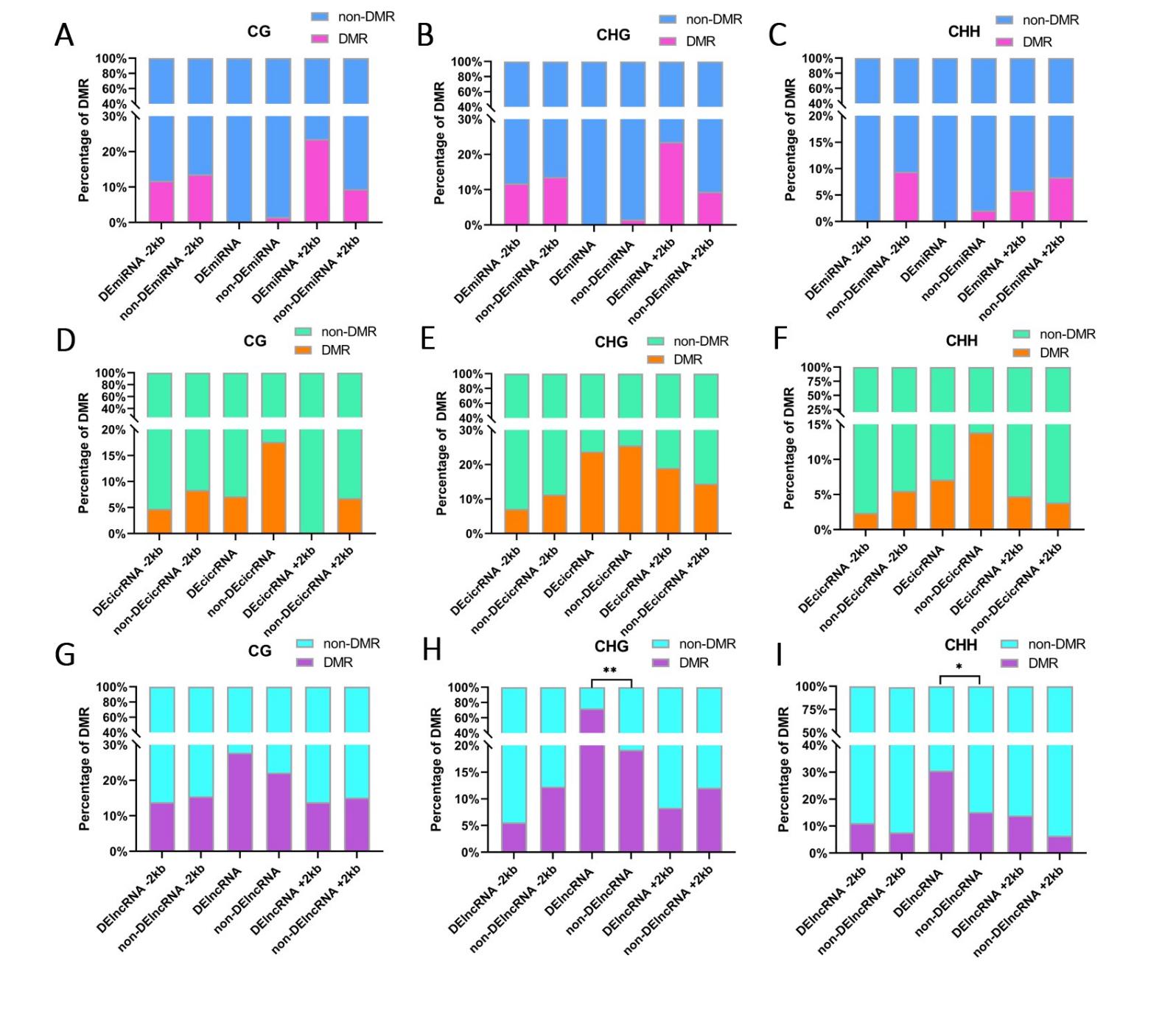


# Figure S8. Histograms showing the percentage of CG-DMR, CHG-DMR, and CHH-DMR in the RNA body and surrounding regions (±2kb) between differentially expressed ncRNAs and non-differentially expressed ncRNAs.

(A-C) figures showing the percentage of DMR in miRNA, (D-F) figures were circRNA, and (G-H) figures were lncRNAs. The significance was tested by chi-square test,

**means p-value <0.01 and * means p-value < 0.05.


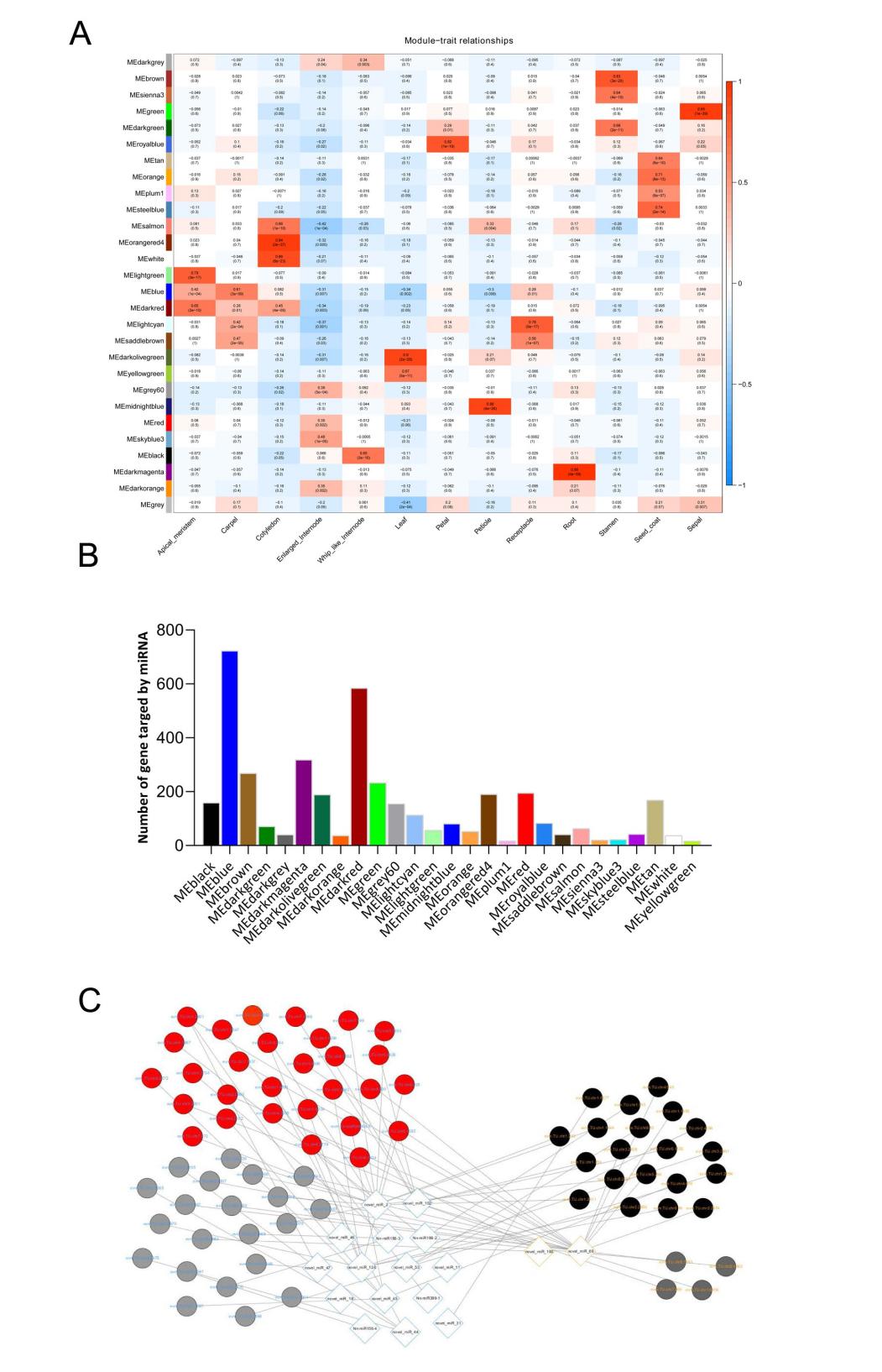


# Figure S9. The weighted gene co-expression networks of different tissue samples in

***N. nucifera*.**

1. The heatmap of WGCNA module-tissue association. Each column corresponds to a tissue. The color of each cell at the row-column intersection indicated the correlation coefficient between the module and tissue type.
2. Distribution of the number of genes targeted by miRNAs in different WGCNA modules.
3. The network of DemiRNAs and their targeted genes are allocated to rhizome internode (enlarge and whip-like) modules. The quadrangles are the DemiRNAs, the quadrangles with blue frames are up-regulated in temperate lotus, and quadrangles with orange frames are up-regulated in tropical lotus. The red and grey circles with blue gene-id are the genes in WGCNA modules that are significantly related to enlarged internode. The black and darkgrey circles with orange gene-id are the genes in WGCNA modules that are significantly related to whip-like internode.

# Figure S10. The maximum likelihood phylogenetic tree of SPL proteins from *Arabidopsis thaliana* (AtSPL), rice (OsSPL) and lotus (NnSPL) using in MEGA X with 1000 replicates. SPLs targeted by miRNA156 are labeled with a red pentacle.


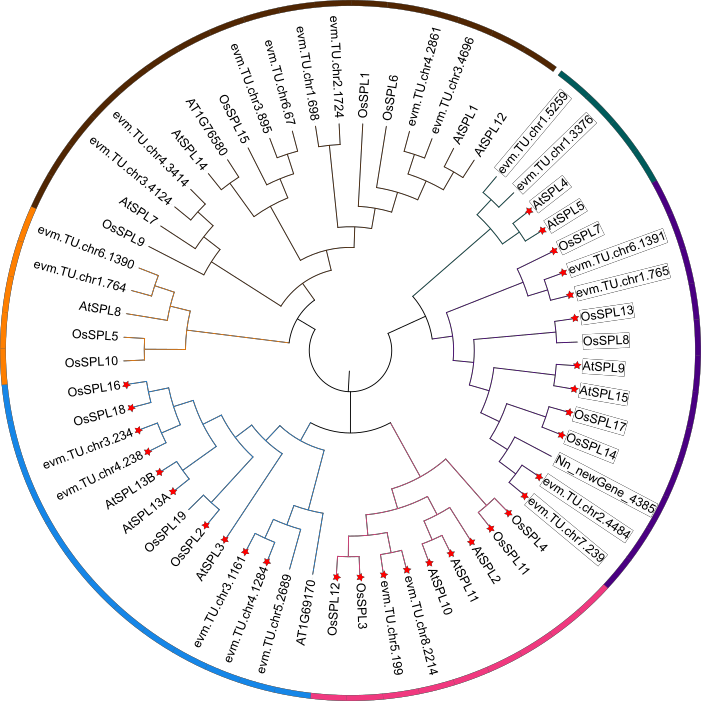


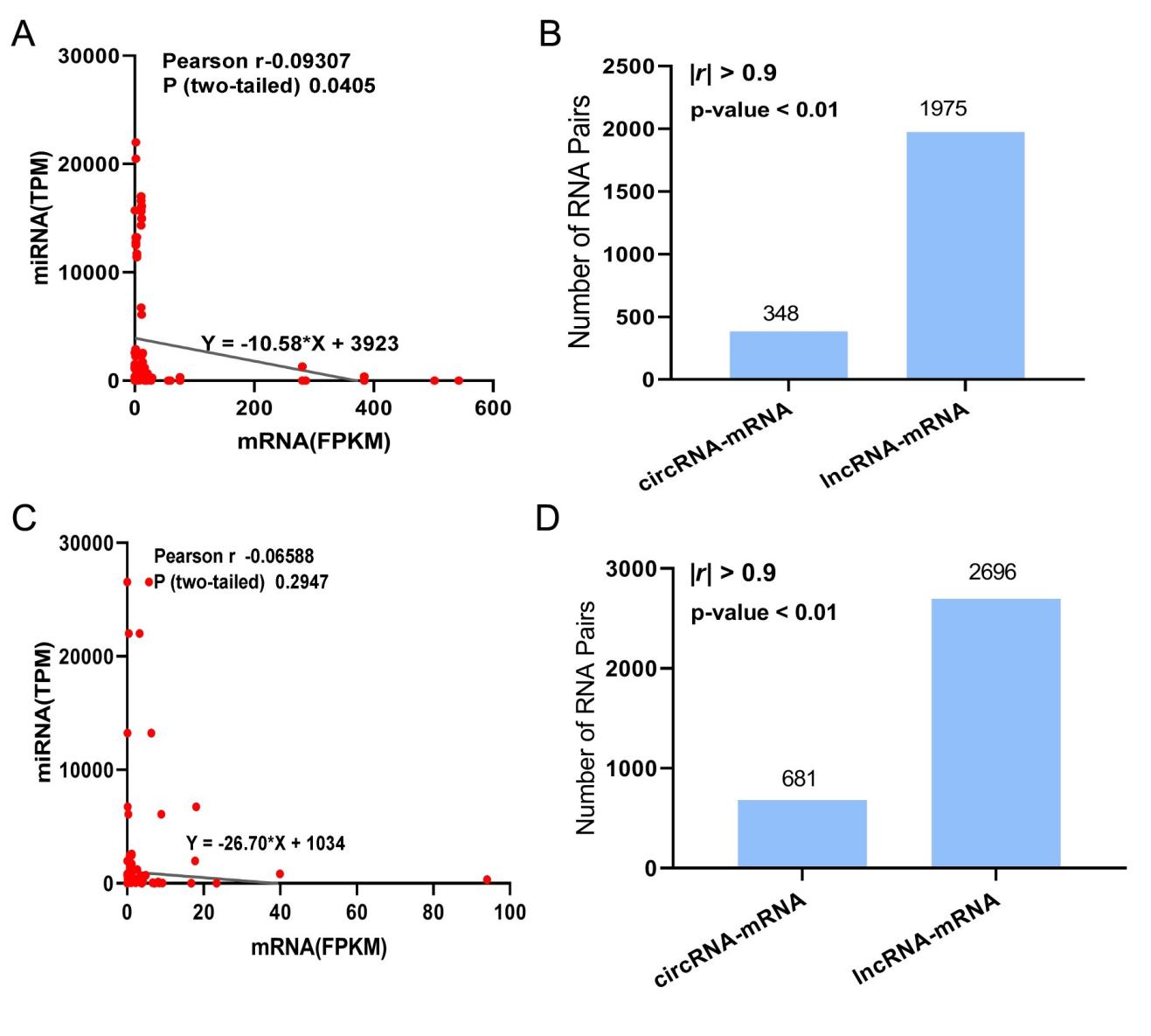


**Figure S11. Dot plot showing the expression correlation between miRNAs and their targeted mRNAs in starch metabolism (A) and auxin signal transduction (C). The number of circRNA-mRNA and lncRNA-mRNA co-expression pairs in starch metabolism (B) and auxin signal transduction (D).**
